# Supplementary material for: Efficient assembly of nanopore reads via highly accurate and intact error correction
Source: Nat Commun. 2021 Jan 4;12:60. doi: 10.1038/s41467-020-20236-7 (PMC7782737; doi:10.1038/s41467-020-20236-7)
Supplement: Supplementary file 1 — Supplementary Information [file 41467_2020_20236_MOESM1_ESM.pdf]

Efficient assembly of Nanopore reads via highly accurate and intact error correction

Ying Chen[1,#], Fan Nie[2,#], Shang-Qian Xie[3,4,#], Ying-Feng Zheng[1,#], Qi Dai[5,#], Thomas Bray[6], Yao-Xin Wang[6], Jian-feng Xing[3,4], Zhi-Jian Huang[7,8,9], De-Peng Wang[10], Li-Juan He[1], Feng Luo[11,*], Jian-Xin Wang[2,*], Yi-Zhi Liu[1,12,*], and Chuan-Le Xiao[1,*]

[1] State Key Laboratory of Ophthalmology, Zhongshan Ophthalmic Center, Sun Yat-sen University #7 Jinsui Road, Tianhe District, Guangzhou, P.R. China
[2] School of Information Science and Engineering, Central South University, Changsha, 410083, China
[3]Key Laboratory of Genetics and Germplasm Innovation of Tropical Special Forest Trees and Ornamental Plants, Ministry of Education, Hainan University, Haikou 570228, China
[4]Hainan Key Laboratory for Biology of Tropical Ornamental Plant Germplasm, College of Forestry, Hainan University, Haikou 570228, China
[5]College of Life Sciences and Medicine, Zhejiang Sci-Tech University, Hangzhou 310018, People's Republic of China
[6]Oxford Nanopore Technologies, Gosling Building, Edmund Halley Road, Oxford Science Park, OX4 4DQ, UK
[7]School of Marine Sciences, Sun Yat-sen University, Guangzhou, Guangdong, People's Republic of China
[8]State Key Laboratory of Biocontrol, Sun Yat-sen University, Guangzhou, Guangdong, People's Republic of China
[9]Southern Marine Sciences and Engineering Guangdong Laboratory (Zhuhai), Sun Yat-sen University, Guangzhou, Guangdong, People's Republic of China
[10]Nextomics Biosciences Co., Ltd
[11]School of Computing, Clemson University, Clemson, SC 29634-0974
[12]Research Units Of Ocular Development And Regeneration, Chinese Academy Of Medical Sciences

*To whom correspondence should be addressed:
Feng Luo. Tel: +01 864 633 6901. Email: luofeng@clemson.edu;
Jian-Xing Wang. Tel: +86 20 87335131. Email: jxwang@mail.csu.edu.cn;
Yi-Zhi Liu. Tel: +86 20 66686996. Email: liuyizh@mail.sysu.edu.cn;
Chuan-Le Xiao: Tel: +86 20 66686996. Email: xiaochuanle@126.com;

#These authors contributed equally to the manuscript as first authors.

Supplementary Note 1：Comparison with assemble-then-correct assemblers

We compared our NECAT assembler with widely used assemble-then-correct assemblers: miniasm[1], Smartdenovo, wtdbg2[2], Flye[3], Raven[4], and Shasta[5] (Supplementary Note 7) using Nanopore data of *E. coli*, *S. cerevisiae*, *A. thaliana*, *D. melanogaster*, *C. reinhardtii*, *O. sativa,* and *S. pennellii*. The miniasm and Shasta didn't have correction step and reported assemblies with a much larger number of mismatches and indels, which were not suitable for the evaluation using QUAST[6]. To make a fair comparison, we ran Racon[7] to improve the accuracy of their assemblies. In addition, the assemblies of Shasta contained many short contigs, we filtered out the contigs $\leq 500bp$ before the evaluation and polishing. We also compared the assemblies of NECAT and Flye on dataset human NA12878 (rel6). In general, assemble-then-correct assemblers run fast but obtain relatively poor assembly results.

As shown in Supplementary Table 5, for relatively less complex genomes, such as *E. coli*, *S. cerevisiae*, all assemblies reported similar NG50 and NGA50, and the running times of NECAT are even less than those of most assemble-then-correct assemblers. For *A. thaliana*, five assemblers, except Smartdenovo and Shasta, reported similar assemblies. For *D. melanogaster*, NECAT reported the best NG50 and NGA50. For more complex genomes, such as *C. reinhardtii*, and *O. sativa*, NECAT reported close to the best NG50 while Flye reported the best in *C. reinhardtii* and Miniasm reported the best in *O. sativa*. The NECAT reported the best NGA50 for *O. sativa* and Flye also reported the best NGA50 for *C. reinhardtii*. For even more complex genome, *S. pennellii*, NECAT reported the best NG50 and NGA50, which were much higher than

those reported by other assemblers. For human NA12878 (rel6), Flye reported higher NG50 and NGA50 than those reported by NECAT, while NECAT reported only one-fourth misassemblies errors. And all assemblers reported similar performance on the number of misassemblies, QV, and gene completeness on the assemblies of *E. coli*, *S. cerevisiae*, *A. thaliana*, *D. melanogaster*, *C. reinhardtii*, *O. sativa,* and *S. pennellii*.

Supplementary Note 2：Validating assemblies from Nanopore reads

We further validated our assemblies by comparing them to reference genomes. The assemblies of the *E. coli*, *S. cerevisiae*, *D. melanogaster*, *C. reinhardtii* were polished by nanopolish[8] and pilon[9]. The assemblies of *A. thaliana* were polished by Arrow[10]. The assemblies of *O. sativa* and Human genomes were polished by Racon[11]. The assemblies of *S. pennellii* genome were polished by pilon (Supplementary Note 10). First, we mapped the assemblies of *E. coli, S. cerevisiae, A. thaliana, C. reinhardtii*, *D. melanogaster*, *O. sativa*, and Human N12878 from Nanopore reads to corresponding reference genomes using MUMmer (v4.0)[12], then evaluated the mapping results using GAGE scripts[13]. Except for the presence of minor structural variations, most assemblies were structurally consistent with reference genomes (Supplementary Figures 6-12). Most assemblies were good collinearity with reference genomes, except the assemblies of *A. thaliana* and *D. melanogaster* generated by wtdbg2, *C. reinhardtii* generated by Canu+smartdenove and smartdenove, and *A. thaliana* generated by Raven. Second, for S. pennellii[14], we mapped the assembly of NECAT to the assemblies of the other pipelines from public paper using MUMmer (v4.0)[12], our assembly was structurally consistent with the assemblies except for the presence of minor structural variations (Supplementary Figure 13) since NG50 of NECAT-generated assembly was much longer than the original reference genome that was generated by Canu+Smartdenvo[14]. The tiling figure also showed that continuity of human N12878 assembly generated by NECAT was better than that generated by Canu (Supplementary Figure 14).

Supplementary Table 6 provided GAGE[13] accuracy metrics for the assemblies of *E.*

coli, S. cerevisiae, A. thaliana, C. reinhardtii, and *D. melanogaster*. The numbers of single-nucleotide polymorphisms (SNPs) and large indels (>10bps) in the genomes assembled by Canu, Canu+Smartdenovo, Smartdenovo, miniasm+Racon, wtdbg2, Flye, Raven, Shasta+Racon, and NECAT were similar. Assemblies reported by NECAT maintained at least 99.30% coverage of their reference genomes.

We then mapped 17,294 annotated genes from *D. melanogaster*[15, 16] onto its three assemblies (Supplementary Note 11). A total of 16,402, 16,438, 16,368, 16,356, 16458, 16,396, 16495, 15796 and 16,412 genes were mapped onto a single contig of assemblies generated using Canu, Canu+smartdenovo, Smartdenovo, miniasm+Racon, wtdbg2, Flye, Raven, Shasta+Racon and NECAT in a single alignment; 15,926, 15,956, 15,979, 15987, 16,084, 16,075, 16121, 15518 and 16,053 of these genes showed over 99% identity. This indicated that the quality of the NECAT assembly was comparable to those of the other pipelines.

Solving repeat regions is the most important task in genome assembly. We first evaluated three assemblies of *D. melanogaster* by comparing the completeness of transposable element (TE) families[17] (Supplementary Note 11). Of the 5,433 annotated TEs from FlyBase, NECAT assembly contained 5,304 TEs, in which 4,001 were aligned perfectly to the reference genome. Flye and wtdbg2 assemblies contained only 3840 and 3831 TEs aligned perfectly to the reference genome, which were less than other assemblies. We then examined two TE families: *roo* and *juan*. Using NECAT assembly, we aligned 134 of the 138 copies in the *roo* family, of which 118 were aligned perfectly. The 11 elements of *juan* family were also aligned perfectly. These results were similar

to those obtained using other pipelines except miniasm+Racon, Raven, and Shasta+Racon. Miniasm+Racon assembly contained 9 perfectly aligned elements of *juan* family, which was the least of all assemblies. Raven and Shasta+Racon assemblies contained only 72 and 69 perfectly aligned elements of *roo* family, which were less than other assemblies (Supplementary Table 7).

We also examined telomeric repeats of 16 chromosomes in the NECAT assembly of *S. cerevisiae* (Supplementary Note 12). We mapped 14 out 16 telomeric repeats to both ends of each chromosome. One telomeric repeat was mapped onto two chromosomes, and the other telomeric repeat was mapped to one end of a chromosome. Our results were similar to those obtained using assemblies generated by other pipelines except wtdbg2. wtdbg2 assembly contained 8 telomeric repeats mapped onto two chromosomes and 5 telomeric repeats mapped to one end of a chromosome (Supplementary Table 8). Both TE of *D. melanogaster* and telomeric repeat of *S. cerevisiae* analyses demonstrated that NECAT could accurately reconstruct repeat sequences.

Supplementary Note 3：Comparison with hybrid pipelines

We also built and evaluated hybrid pipelines to show the correctness and effectiveness of the correction step and the assembly step of NECAT (Supplementary Note 7). We combined either the correction step of NECAT with the assembly steps of Canu, Smartdenovo, and Flye, or the correction step of Canu with Smartdenove, Flye and the assembly step of NECAT. Then, we used those hybrid assembly pipelines to assemble datasets of *E. coli*, *S. cerevisiae*, *A. thaliana*, *D. melanogaster* and *C. reinhardtii*. The performances of the hybrid pipelines are shown in Supplementary Table 9.

All pipelines reported similar NG50 and NGA50 for *E. coli* and *S. cerevisiae*, except NECAT+Flye reported as smaller NGA50 due to one more mis-assembly error. For *A. thaliana,* NECAT+Flye reported the best NG50 and NGA50, while Canu+S, NECAT+Canu, and NECAT reported close to the best NG50 and NGA50. For *D. melanogaster*, NECAT reported the best NG50, while Canu+S, Canu+NECAT, and NECAT+S reported close to best results. The NECAT+S reported the best NGA50 for *D. melanogaster*. For *C. reinhardtii*, NECAT+Flye reported the best NG50 while NECAT reported the close to the best one. The Canu+Flye reported the best NGA50 for *C. reinhardtii*. Our comparison showed that NECAT reported consistent performance on the assemblies of all five genomes, while the performances of other hybrid pipelines were not stable.

Moreover, we used the NECAT+Flye to ensemble human NA12878 (rel6). We obtained an assembly with 19% higher NG50 and slightly higher NGA50 comparing to

those of the assembly from Flye. The number of misassembly in the assembly of NECAT+Flye was also significantly less than those in the assembly of Flye. These results implied that the “correct-then-assembly” approach may be more appropriate for assembling large complex genomes.

Supplementary Note 4: Cell culture and sequencing materials

Datasets for eight species (*E. coli*, *S. cerevisiae*, *A. thaliana*, *D. melanogaster*, *C. reinhardtii*, *O. sativa*, *S. pennellii* and *H. sapiens*) were used to train and test our algorithm. Among these, four datasets (*S. cerevisiae*, *C. reinhardtii*, *O. sativa Japonica Group*, and retinoblastoma cell line WERI) were cultured and sequenced using MinION / PromethION platform from Oxford Nanopore in our laboratory; detailed culture conditions are described in the following text.

S. cerevisiae **w303 culture:** *S. cerevisiae* strains w303 were cultured in Yeast Extract Peptone Dextrose (YPD) broth used as a complete medium for yeast growth. YPD medium, which contained 1 L of deionized water to 20 g bacto peptone, 10 g yeast extract, and 20 g dextrose, was sterilized by autoclaving for 20 min at 15 psi (1.05 kg/cm^2), and was stored at room temperature. Yeast cells were cultured at 30°C in a shaking incubator at 300 rpm for 24 to 36 hours.

C. reinhardtii **culture:** High-quality genomic DNA was extracted from *C. reinhardtii* cultured under mixotrophic (constant light) or heterotrophic (constant dark) conditions in Tris-Acetate-Phosphate (TAP) medium during the pre-stationary phase. Samples of wild-type strain CC-1690 were placed in an intelligent temperature and illumination incubator under 4~6°C and 20~30 μE/(m^2·s) light intensity. The naturally synchronized cells were induced using a 12 h/12 h light/dark cycle.

Culture of O. sativa Japonica Group： The seeds of O. sativa Japonica Group (Janponica Nipponbare) were sterilized, immersed in deionized water and germinated in the dark for 3 days. After germination, seedlings were transplanted into plastic pots filled with commercial substrate (PINDSTRUP, Denmark), and kept in a growth chamber at26/22° C ±1°C day/night temperature and light intensity of 600 μmolm^{-2}s^{-1}. Four-weeks old seedlings were harvested for DNA isolation.

Culture of retinoblastoma cell line WERI: The human retinoblastoma cell line WERI was cultured in RPMI 1640 (Gibco Company, USA) supplemented with 20% fetal bovine serum (Biological Industries, USA). Cell cultures were incubated at 37°C and

5% CO_2, and media were replaced every 3~4 days. Cultures were maintained using centrifugation and resuspension in fresh medium, or media replacement after cell aggregates precipitated at the bottom of the flask. Cells were grown in suspension at a concentration of 10^5~10^6 cells/ml.

Supplementary Note 5: DNA extraction and purification

***S. cerevisiae* w303:** *S. cerevisiae* w303 cells were washed twice using phosphate-buffered saline (PBS) and collected by centrifugation at 4,000 rpm for 5 min. Samples were: (i) lysed in buffer with 1 ml lysozyme TLB and 20 µl RNase A (20 mg/ml), and then incubated for 1 h at 37°C; (ii) treated with 20 µl Proteinase K for 1.5 h at 50°C; (iii) purified with 1 volume phenol, 0.5 volume phenol-chloroform (1:1 by volume), 3 volume ice-cold absolute ethyl alcohol at 4,500 rpm for 10 min; (iv) washed in 80% ice-cold ethanol twice, collected by centrifugation (12,000 rpm, 15 min, 4°C), and eluted in 100 µl elution buffer(EB; 10 mMTris hydrochloride [pH 8.0]).

***C. reinhardtii* and *O. sativa Japonica Group*:** High-molecular-weight (HMW) DNA was isolated from *C. reinhardtii* cc1690 and *O. sativa Japonica Group* using the CTAB method. Briefly, about 0.2 g samples were re-suspended in 1 ml CTAB buffer containing 2% β-mercaptoethanol, incubated at 65°C for 30 min, and then centrifuged at 8,000 rpm for 5 min. The suspended nuclei were purified twice with chloroform-isoamyl alcohol (24:1 by volume) and once with 0.7 volume isopropyl alcohol at -20°C for 1 h. DNA precipitates were washed in ice-cold 75% ethanol twice, collected by centrifugation (12,000 rpm at 15 min and 4°C), dried under vacuum, and re-suspended in 100 ul EB[18] (10 mM Tris hydrochloride [pH 8.0]).

Retinoblastoma cell line WERI: 1 x 10^7 frozen cells were lysed with 800 µl TEN Buffer, 100 µl 20% sodium dodecyl sulphate (SDS), and 100 µl proteinase K. This mixture was incubated at 56°C for 2 hours, purified with phenol-chloroform-isoamyl alcohol (25:24:1 by volume) and chloroform-isoamyl alcohol (24:1 by volume), and precipitated using 0.7 volume isopropyl alcohol at -20°C for 40 min. DNA precipitates were collected by centrifugation (12,000 rpm at 15 min and 4°C), washed twice in ice-cold 80% ethanol, dried under vacuum, re-suspended in 100 ul EB (10 mMTris hydrochloride [pH 8.0]), and combined with 2 µl RNase A (100 mg/ml) to cleave the RNA. To acquire high-quality DNA for the three datasets mentioned above, an

additional purification step was performed using 0.8 volume magnetic beads from an AMPure XP kit (#A63882, Agencourt) according to the manufacturer's instructions.

Supplementary Note 6: Nanopore whole genome sequencing and base-calling

***S. cerevisiae* w303:** Sequencing libraries were constructed using a Ligation Sequencing Kit 1D (SQK-LSK108, Oxford Nanopore, UK) according to the manufacturer's instructions. Then, 5 µg high-molecular-weight genomic DNA was fragmented using g-TUBE (#520079, Covaris) centrifugation (conducted twice at 1,400 g for 2 min). Libraries were prepared according to the manufacturer's instructions. Briefly, NEBNext Ultra II End-Repair/dA-tailing module (#E7546, NEB) was used to end-repair and dA-tail the DNA fragments. Then, each dA-tailed sample was tethered to 1D adapter using NEBBlunt/TA Ligase Master Mix (#M0367, NEB). The prepared DNA library was loaded into R9.4 flow cells and sequenced on MinION sequencers (Oxford Nanopore). The raw data, collected in this experiment, were obtained as fast5 files after conversion of electrical signals into base calls via Albacore 1.1.0 (Oxford Nanopore Technologies).

***C. reinhardtii, O. sativa* and retinoblastoma cell line WERI:** Large insert-size libraries of *C. reinhardtii, O. sativa* and retinoblastoma WERI cells were created according to the manufacturer's protocols (Oxford Nanopore, UK). Briefly, 5 µg genomic DNA was sheared into ~20-30 kb fragments using g-TUBE (#520079, Covaris) centrifugation (twice at 1,400 g for 2 min) and size-selected (>8-10 kb) by Blue Pippin (Sage Science, MA) using a marker started at 5-12 min (0.75% DF Marker S1 High-Pass 6-10kb vs3) to ensure the removal of small DNA fragments. Genomic DNA libraries were prepared using a Ligation sequencing 1D kit (SQK-LSK109, Oxford Nanopore, UK). End-repair and dA-tailing of DNA fragments were performed using an Ultra II End Prep module (#E7546, NEB) according protocol recommendations. Each dA-tailed sample was tethered to 1D adapter using a Quick Ligation Module (#E6056, NEB). The prepared DNA library was loaded into a FLO-PRO002 flow cell and sequenced on PromethION sequencers (Oxford Nanopore, UK). The raw data collected in this experiment was obtained as fast5 files after conversion of electrical signals into base calls via guppy 2.0.8 (Oxford Nanopore, UK).

Supplementary Note 7：Statistics for Nanopore datasets

To evaluate the performance of NECAT, we collected eight datasets for *E. coli, S. cerevisiae, A. thaliana*[19], *D. melanogaster*[20], *C. reinhardtii, O. sativa*, *S. pennellii*, and *H. sapiens*[21] (NA12878). Details can be found in Supplementary Table 1. Among these eight datasets, data on *E. coli, A. thaliana, D. melanogaster*, *S. pennellii*, and *H. sapiens* (NA12878) were available from public websites, and the other two datasets were generated using our in-house sequencing. Their corresponding short-reads datasets of Next Generation Sequencing (NGS) were collected from the related projects at NCBI. All SRA files were converted to fastq files using an SRA Toolkit[22] (https://www.ncbi.nlm.nih.gov/sra/docs/toolkitsoft/) from NCBI. Raw long-read files in fastq or fasta format were used as input files for these assembly pipelines. Nanopore fast5 format files and NGS fastq format files were used as input files for Nanopolish[8] and Pilon[9], respectively.

The results of basic statistical analysis for raw long reads (LRs) are shown in Supplementary Table 2. Seqkit (v0.8.0)[23] was used to directly calculate "Base Counts," "LR Count," "N50 Length," and "Mean Length." We then used scripts to calculate "N75 Length" and "N25 Length" based on results obtained using Seqkit. N75, N50, and N25 represented sequence lengths sorted in descending order when the accumulated length of the sequence reached 75, 50, and 25% of the total number of bases ("Base Counts"), respectively. Finally, we divided "Base Counts" by general genome size (*E. coli*: 4,600,000 base pairs [bp], *D. melanogaster*: 137,000,000 bp, *A. thaliana*: 125,000,000 bp, *S. cerevisiae*: 12,000,000 bp, *C. reinhardtii*: 120,000,000 bp, *O. sativa*:370,000,000, *S. pennellii:* 886,000,000 and *H. sapiens*: 3,000,000,000 bp) of the corresponding species to calculate coverage. Among these eight datasets, *A. thaliana* and *H. sapiens* datasets showed very low coverage (27X and 38X), while the other datasets showed more than 50X coverage.

N25 and N75 lengths were calculated using the following shell scripts:

```
ecoli=pathto/E.coli.fasta
yeast=pathto/w303.fastq
```

```
dro=pathto/dro.fastq
arab=pathto/arab.fastq
cre=pathto/cre.fastq
human=pathto/human.fastq
for i in ${ecoli ${yeast} ${dro} $22 ${cre} ${human};
do
seqkit fx2tab -j 10 -l -n -i -H ${i} | cut -f 4 | sed'1d' | sort -rn>
${i}.lenth.txt
seqkit stats -j 10 -a ${i} >>statistic.txt
all=$(awk'BEGIN{n=O}{n=n+$1}END{print n}' ${i}.lenth.txt)
echo"N75">>statistic.txt
awk'BEGIN{n=O}{if (n>="'$all'"*0.75){print $1;}n=n+$1;}' ${i}.lenth.txt |
head -n 1 >>statistic.txt
echo"N25">> statistic.txt
awk'BEGIN{n=O}{if (n>="'$all'"*0.25){print $1;}n=n+$1;}' ${i}.lenth.txt |
head -n 1 >> statistic.txt
done
```

Supplementary Note 8：Error analysis of Nanopore raw reads

Raw noisy LRs were corrected by mainstream consensus algorithms using the following steps: (1) building a multiple sequence alignment (MSA) from pairwise alignments and (2) choosing the correct base from MSA columns. FalconSense[24], Recon[11], Nanocorrect[8], and Dacoordare[25] are widely-used correction algorithms for Nanopore raw long reads. FalconSense uses the tagging and sorting approach to construct a consensus sequence based on consistent base-level and partial-order alignment. FalconSense and Recon were adopted for Nanopore sequence correction by fine-tuning parameters. Nanocorrect used a correction method similar to DAGCon[26], which encoded the MSA as a partial-order alignment with a directed acyclic graph (DAG). Dacoord resolves the corrected bases of repeated regions using a local de-Bruijn assembly-map algorithm. However, the accuracy and integrity of Nanopore corrected sequences produced by the above methods remained limited.

To determine whether the existing correction algorithms were feasible for correction of Nanopore raw reads, we needed to obtain the features of sequencing errors in Nanopore LR data. First, we analyzed error distribution of Nanopore datasets for *E. coli, S. cerevisiae*, *A. thaliana*, *D. melanogaster, C. reinhardtii*, *O. sativa*, *S. pennellii*, and *H. sapiens* (NA12878). We used reference genomes as standard sequences. Raw long reads of these Nanopore datasets were aligned using minimap2[27] against their corresponding reference genomes (Supplementary Table 1). Then, we statistically analyzed error distribution of each dataset according to the mismatched results.

Our results indicate that sequencing error rate of Nanopore reads was as high as 10-30% and broadly distributed（Figure 1A and Supplementary Table 3). We also found that the error rates of different positions differed broadly in each read, and the reads were generally present as high-error-rate subsequences (HERS), whose sequencing error rates were > 50% in these subsequences (Figure 1B). These sequencing error characteristics differed greatly from those in PacBio datasets (Figure 1). These results highlight the necessity of developing a specific consensus algorithm for Nanopore raw

data.

We used the following scripts for aligning Nanopore datasets to their corresponding reference genomes:

```
minimap2 -t 20 -ax map-ont ${ref_fasta} ${reads_fasta} > ${species}_aln.sam
```

The error bases of all mapped reads were extracted and counted using the following scripts:

```
awk'{print $3"\t"$4"\t"$10"\t"$12}' ${species}_aln.sam
|awk'{split($4,a,":");print $1"\t"$2"\t"length($3)"\t"a[3]}' | awk'$3>100
{print $0}' | awk'/^NC/ {print $0}'> ${species}_stat_clean.txt
```

The distributions of sequencing errors for the six datasets were plotted using the following R scripts (Figure 1A):

```
ecolia=read.table(file="ecoli_stat_clean.txt")
yeast=read.table(file="yeast_stat_clean.txt")
arab=read.table(file="arab_stat_clean.txt")
dro=read.table(file="dro_stat_clean.txt")
yizao=read.table(file="yizao_stat_clean.txt")
human3=read.table(file="human_stat_clean.txt")
rice=read.table(file=" rice_stat_clean.txt")
tomato=read.table(file=" tom_stat_clean.txt")
for(i in 1:8)
{
if(i==1) {ecoli=ecolia} ; if(i==2) {ecoli=yeast} ; if(i==3) {ecoli=arab};
if(i==4) {ecoli=dro}; if(i==5) {ecoli=yizao}; if(i==6) {ecoli=human}; if(i==7)
{ecoli=rice}; if(i==8) {ecoli=tomato}
ecoli_n=numeric()
ecoli_s=cbind(ecoli,ecoli[,4]/ecoli[,3])
ecoli_t=dim(ecoli_s)[1]
for(j in 1:50){
  if(j==1){
    ecoli_n[1]=length(ecoli_s[ecoli_s[,5]<=0.01,5])/ecoli_t
  }
  else{
    pos2<-j/100
    pos1<-(j-1)/100
    ecoli_n[j]=length(ecoli_s[ecoli_s[,5]<=pos2 & ecoli_s[,5]>pos1,5])/ecoli_t
  }
}
if(i==1) {ecolin=ecoli_n}; if(i==2) {yeastn=ecoli_n};if(i==3) {arabn=ecoli_n}
if(i==4) {dron=ecoli_n}; if(i==5) {yizaon=ecoli_n}; if(i==6) {human3n=ecoli_n};
```

```
if(i==7) {ricen=ecoli_n}; if(i==8) {tomaton=ecoli_n}
}
pdf("read-error-distribution-fcraction.pdf")
plot(ecolin~c(1:50),ylab="Fraction of error rate (%)",xlab="Error rate
(%)",ylim=c(0,0.20),col="darkgreen",type="l",axes=F,lwd=3,lty=1)
lines(yeastn~c(1:50),col="darkblue",lwd=3,lty=1)
lines(arabn~c(1:50),col="coral4",lwd=3,lty=1)
lines(dron~c(1:50),col="darkorange3",lwd=3,lty=1)
lines(yizaon~c(1:50),col="firebrick2",lwd=3,lty=1)
lines(human3n~c(1:50),col="yellow4",lwd=3,lty=1)
lines(ricen~c(1:50),col="chartreuse",lwd=3,lty=1)
lines(tomn~c(1:50),col="darkviolet",lwd=3,lty=1)
legend(25,0.18,legend=c("E.coli","Yeast","A.thaliana","D.melanogaster","C.re
inhardtii","Human"),col=c("darkgreen","darkblue" ,"coral4" ,"darkorange3","f
irebrick2","yellow4" ,"chartreuse","darkviolet"),lty=1,cex=1.5,box.lty=0,lwd
=2)
axis(2,at=c(0,0.05,0.10,0.15,0.20),labels=c("0","5","10","15","20"),las=1,lw
d=1,tick=T)
axis(1,at=c(0,10,20,30,40,50),labels=c("0","10","20","30","40","50"),las=1,l
wd=1,tick=T)
dev.off()
```

To further understand if there was a bias for sequencing errors among different genome positions, we calculated sequencing-error distribution for all mapped reads on different genome locations (Supplementary Figure 1). The scripts were:

```
awk'{print $1"\t"$2"\t"$2+$3"\t"$4/$3}' ${species}_stat_clean.txt |sort -
k1,1 -k2,2n > ${species}.sorted.bed
refgenome=~/xsq/project/ONT_correct/distribution/data/${species}.fa
line=$(wc -l $refgenome |awk'{print $1}')
awk -v lin=$line '{if(NR!=lin&&/^>/) {print $1"\t"NR;tmp=$1}
if(NR==lin) {print tmp"\t"NR}}' $refgenome\
|awk'NR==1 {tmp1=$1;tmp2=$2 }
NR!=1 {print tmp1"\t"tmp2"\t"$2"\t"($2-tmp2-1)*80; tmp1=$1; tmp2=$2}'
|awk'{len=$4/10000; for(i=1;i<=len;i++) {print $1"\t"(i-
1)*10000+1"\t"10000*i}}' |awk'{split($1,a,">"); print
a[2]"\t"$2"\t"$3 }'>${species}_10000.bed
export PATH=$PATH:/software/bedtools2/bin
bedtools intersect -loj -a ${species}_10000.bed -b
${species}.sorted.bed>position_{species}.txt
```

To further understand the different error distribution in each read, we extracted each raw read, and calculated the mismatch and indel base number in a region having a length >500 bp (Figure 1B). The scripts were:

```
awk '{tmp=0; for (i=1;i<length($6);i++) {st=substr($6,i,1); if(st~/[0-9]/) 
{ss=ss""st}
if(st=="M") {mn=mn+ss;mi=mi+ss;tn=tn+ss;ss=""}
if(st=="D") {tn=tn+ss;ss=""}
if(st=="I") {tn=tn+ss;mi=mi+ss;ss=""}
if(st~/[A-Za-z]/ ) {ss=""}
if(mi>500&&st=="M") {for(j=mi;j>500;j=j-500){re=re"_"(mn+500-j)/tn;tn=j-
500;mn=j-500;mi=j-500}}
if(mi>500&&st=="I") {for(j=mi;j>500;j=j-500){re=re"_"mn/tn;tn=j-500;mi=j-
500;mn=0 }}
if(mi==500){re=re"_"mn/tn;tn=0;mi=0;mn=0}
}; print re}'  ERR2173373.21178.sam | sed s/_/'\n'/g | awk 'NR>1{print (NR-
2)*500"-"(NR-1)*500"\t"1-$1}' > stat_500.res
```

Then, error subsequences of 500 bp in each read were plotted and beautified by Excel and Adobe Illustrator.

The high error rate subsequences (HERS) of eight datasets were extracted as similarly as each read. The scripts were:

```
awk -v var=10 'length($10)>var*1000 {tmp=0; for (i=1;i<length($6);i++) 
{st=substr($6,i,1); if(st~/[0-9]/) {ss=ss""st}
     if(st=="M") {mn=mn+ss;tn=tn+ss;ss=""}
      if(st=="D") {tn=tn+ss;ss=""}
     if(st=="I") {tn=tn+ss;ss=""}
     if(st~/[[:alpha:]]/ ) {ss=""}
     if(tn>500) {if(mn/tn<0.50) {tmp=1;break}; mn=0;tn=0 }
    }if(tmp==1) {tsum=tsum+1;;mn=0;tn=0};
     print NR,length($10),tmp}
    '  mutilsam/tom_raw_aln$i.sam > stat_500.res.txt
```

After extracting high error rate subsequences, HERS distributions for the six datasets were plotted using the following R scripts (Figure 1C):

```
ecoli=read.table(file="ecoli/stat_500.res.txt")
yeast=read.table(file="yeast/stat_500.res.txt")
arab=read.table(file="arab/stat_500.res.txt")
dro=read.table(file="dro/stat_500.res.txt")
yizao=read.table(file="yizao/stat_500.res.txt")
human=read.table(file="human/stat_500.res.txt")
```

```
rice=read.table(file="rice/stat_500.res.txt")
tomato=read.table(file=tomato/stat_500.res.txt")
result=matrix(,8,41)
for(j in 1:8)
{if(j==1)  {a=ecoli}  ;  if(j==2){a=yeast};  if(j==3){a=arab};  if(j==4){a=dro};
if(j==5){a=yizao}; if(j==6){a=human}; if(j==7){a=rice}; if(j==8){a=tomato};
 for(i in 1:41)
 {  usum=a[a[,2]>=(i+9)*1000&a[,3]==1,3]
    tsum=a[a[,2]>=(i+9)*1000,3]
    if (length(usum)>=500){ result[j,i]=length(usum)/length(tsum)}}
}
tmp1=result[1,][!is.na(result[1,1:41])];tmp2=result[2,][!is.na(result[2,1:41
])];tmp3=result[3,][!is.na(result[3,1:41])];tmp4=result[4,][!is.na(result[4,
1:41])];tmp5=result[5,][!is.na(result[5,1:41])];tmp6=result[6,][!is.na(resul
t[6,1:41])];tmp7=result[7,][!is.na(result[7,1:41])];tmp8=result[8,][!is.na(r
esult[8,1:41])]
pdf("HER length.pdf")
plot(result[1,1:length(tmp1)]~c(1:length(tmp1)),type="l",axes=F,lwd=3,lty=1,
col="darkgreen",  ylim=c(0,0.5),  xlim=c(1,41),  xlab="Read  length(kb)",  ylab=
"Fraction of reads with HER")
axis(2,at=c(0,0.1,0.20,0.3,0.4,0.5),labels=c("0","10","20","30","40","50"),
las=1,lwd=1,tick=T)
axis(1,at=c(1,11,21,31,41),labels=c("10","20","30","40","50"),las=1,lwd=1,ti
ck=T)
lines(result[2,1:length(tmp2)]~c(1:length(tmp2)),col="darkblue",lwd=3,lty=1)
lines(result[3,1:length(tmp3)]~c(1:length(tmp3)),col="coral4",lwd=3,lty=1)
lines(result[4,1:length(tmp4)]~c(1:length(tmp4)),col="darkorange3",lwd=3,lty
=1)
lines(result[5,1:length(tmp5)]~c(1:length(tmp5)),col="firebrick2",lwd=3,lty=
1)
lines(result[6,1:length(tmp6)]~c(1:length(tmp6)),col="yellow4",lwd=3,lty=1)
lines(result[7,1:length(tmp7)]~c(1:length(tmp7)),col="chartreuse",lwd=3,lty=
1)
lines(result[8,1:length(tmp8)]~c(1:length(tmp8)),col="darkviolet",lwd=3,lty=
1)
legend(3,0.5,legend=c("E.coli","Yeast","A.thaliana","D.melanogaster",
"C.reinhardtii","Human"),col=c("darkgreen","darkblue"  ,"coral4"  ,"darkorange
3","firebrick2",  "yellow4"  ,"chartreuse","darkviolet"),lty=1,cex=1.2,box.lty
=0,lwd=2)
dev.off()
```

Supplementary Note 9: Performance of error correcting algorithms

Due to the high sequencing error discrepancy between Nanopore raw reads and PacBio raw reads (Figure1 and Supplementary Note 5), the existing correction methods developed specifically for PacBio reads are unsuitable for Nanopore data. To date, there is no correction method that fully accounts for characteristics of sequencing errors occurring in Nanopore data.

In this study, we developed a novel progressive two-step error correction algorithm called NECAT with adaptive candidate-read selection for Nanopore raw reads. In order to validate the rationality and reliability of our novel algorithm, we examined the performance of NECAT in correcting the eight datasets described above (Supplementary Table 1). For comparison, we also evaluated the accuracy of reads corrected by Canu[28], another widely-used correction tool for Nanopore raw reads. Specifically, for each dataset, we calculated error rates of: the raw dataset, corrected reads after step one in NECAT, corrected reads after step two in NECAT, and reads corrected by Canu[28]. For this, we mapped the four datasets to the reference using minimap2[27] as described in Supplementary Note 5. Then, results of the alignment were used to calculate error distribution. Error rates were grouped by 1, 2, 3, 4, 5, 6, 7, 8, 9, 10, 10-15, 15-20, 20-25, 25-30, and 30-100%, and results are listed in Supplementary Table 4. The following scripts were used:

```
for i in 1 2 "canu" "raw"
do
cd $i
awk'{print $3"\t"$4"\t"$10"\t"$12}' ${species}_${i}_aln.sam |awk'{split($4,
a, ":"); print $1"\t"$2"\t"length($3)"\t"a[3]}' | awk'$3>100 {print $0}' |
awk'/^chr/ {print $0}'> ${species}_stat_clean_$i.txt
Rscript correct_stats_ref.r ${species}_stat_clean_$i.txt
"correct_stat.result"
cd ..
done
```

In each raw dataset, we then analyzed a HERs region having a length >500 bp. For mapped reads in each of the four datasets, we evaluated raw reads, corrected reads after first correction of NECAT, corrected reads after second correction of NECAT, and

corrected read output by Canu. Considering canu only selects the longest 40x for correction by default, we extracted the sub-dataset with equal coverage from the raw dataset, corrected reads after step one in NECAT and corrected reads after step two in NECAT. The scripts were:

```
###species can use eight species, we take e.coli for example
species=ecoli
size=`ls -ltr ecoli_canu.fasta | awk '{print $5}'`
for i in 1 2 "raw"seqfasta= ecoli_$i.fasta
awk 'NR%2==1 {tmp=$1}NR%2==0 {print tmp"_XSQ_"$0"\t"length($0)}'\
${seqfasra} | sort -nr -k 2 | awk -v si=$size 'tmp=tmp+$2\
 {if(tmp<si){print $0} if(tmp>=si) exit}' | \
awk '{split($1,a,"_XSQ_");print a[1]"_XSQ_"$2"\n\r"a[2]}' >
rice${i}_filter.fasta
```

In order to calculate the number of gaps, we generated alignment paf files using minimap2. The scripts were:

```
reffasta=ecoli_k12_genomic.fna
for i in 1 2 "raw"
do
echo $i
mkdir -p ~/alignment/minimap2/$species/$i
cd~/alignment/minimap2/$species/$i
seqfasta= /data/$i/ecoli$i_filter.fasta
minimap2 -t 20 -x map-ont ${reffasta} ${seqfasta} >${species}_${i}_aln.paf
done
minimap2 -t 20 -x map-ont ${reffasta} ecoli_canu.fasta > ecoli_canu_aln.paf
```

For raw reads, we extracted all the reads with gaps >500 bp, and counted the number of HERs regions using the following scripts:

```
awk'{print $6"_"$1"\t"$3"\t"$4"\t"$2}' ${species}_raw_aln.paf> \
${species}_raw_bed.txt
sort -k1,1 -k2,2n ${species}_raw_bed.txt |uniq>in.sorted.bed
bedtools merge -iin.sorted.bed -d 500 | awk'{print $1}' |uniq -d
|awk'{split($1,a,"_"); {print a[3]"\t"1"\t"10000}}'>
${species}_gap_read_name.txt
wc -l  ${species}_gap_read_name.txt
```

For these raw reads with gaps, we re-calculated the HERs region number in these reads after first correction of NECAT, after second correction of NECAT, and after correction of Canu. For outputted corrected reads from Canu, we extracted the reads having a

HERs region >500 bp and counted the number of these regions using the following scripts:

```
awk'split($1,a,"_") {print a[1]"\t"$3"\t"$4"\t"$6}' ${species}_canu_aln.paf> ${species}_canu_bed.paf
sort -k1,1 -k2,2n ${species}_gap_read_name.txt |uniq |bedtools merge -i - -d 500 |awk'{print $1}' |uniq -d >read_gap.result.final
wc -l read_gap.result.final
```

For corrected reads produced by step one and step two in NECAT, reads having a HERs region >500 bp were extracted using the following scripts:

```
for i in "ecoli" "yeast" "dro" "ara" "yizao" "human" "rice" "tomato"
do
for j in 1 2
do
cd ${i}/${j}
awk'{split($1,a,"_\\(");print a[1]"\t"$3"\t"$4"\t"$6}' ${i}_${j}_aln.paf> ${i}_${j}_bed.paf
cd ../..
done
done
```

Finally, the gap number was counted by:

```
sort -k1,1 -k2,2n ${species}_1_bed.paf |uniq |bedtools merge -i - -d 500 |awk'{print $1}' |uniq -d |wc -l
```

The number of HERS regions with large gaps > 500 bp in each raw and corrected dataset can be found in Table 1.

Supplementary Note 10: Comparison of assembly pipelines

We compared the quality of assembly results and running time for Canu (v1.8)[28], Canu (v1.8)+Smartdenovo (5cc1356)[29], Smartdenovo (5cc1356), miniasm (1552e6f)[1], wtdbg2 (v2.5)[30], Flye (2.6)[31], Raven(1.1.5)[4], Shasta(0.4.0)[5], and NECAT (47c6c23) pipelines. Running time was recorded from the log files. All assemblers ran on a 4-core 24-thread Intel(R) Xeon(R) 2.4 GHz CPU (CPU E7-8894[v4]) machine with 3 TB of RAM; the OS was Centos 7.3 64-bit (Linux). The eight datasets (*E. coli, S. cerevisiae, A. thaliana*, *D. melanogaster*, *C. reinhardtii*, *O. sativa*, *S. pennellii* and *H. sapiens*) composed of Nanopore long reads were assembled by the pipelines. The *de-novo* genome assemblies of eight datasets and results of statistical analyses are shown in Table 2 and Supplementary Table 5.

Canu pipeline was run as:

```
echo Start: $(date "+%Y-%m-%d %H:%M:%S")
canu -p $genomeName -d $genomeName genomeSize=$genomeSize maxMemory=1000
maxThreads=$threads useGrid=false -nanopore-raw input.fastq
echo End: $(date "+%Y-%m-%d %H:%M:%S")
```

where *$genomeName* was set to *E. coli, S. cerevisiae, A. thaliana*, *D. melanogaster*, *C. reinhardtii*, *O. sativa* and *S. pennellii,* respectively, and *$genomeSize* was set to 4.8M, 13M, 130M, 130M, 120M, 400 M and 1G, respectively. $theads was set to 32 for *E. coli, S. cerevisiae, A. thaliana*, *D. melanogaster* and *C. reinhardtii* and 64 for *O. sativa* and*S. pennellii.*

For Canu+smartdenovo pipeline, the output file *$genomeName.correctedReads.fasta* from the Canu pipeline was used as input file to the Canu+smartdenovo pipeline; the script was as follows:

```
echo Start: $(date "+%Y-%m-%d %H:%M:%S")
smartdenovo.pl -p $genomeName -t $threads -c 1
$genomeName.correctedReads.fasta > $genomeName.mak
make -f $genomeName.mak
echo End: $(date "+%Y-%m-%d %H:%M:%S")
```

For the Flye pipeline, we used the following script:

```
echo Start: $(date "+%Y-%m-%d %H:%M:%S")
flye --nano-raw input.fastq --out-dir $genomeName --genome-size $genomeSize
```

```
--threads $threads
echo End: $(date "+%Y-%m-%d %H:%M:%S")
```

Flye failed to run on raw reads of *E. coli* and *C. reinhardtii*, for the input files contained malformated reads and duplicate reads. We used the following scripts to filter the raw reads before running Flye. For *E. coli*, the script was:

```
fsa_rd_tools longest --base_size 0 --discard_illegal_read --ifname inputfile
--ofname outputfile
```

`fsa_rd_tools` was a tool in NECAT pipeline.

For *C. reinhardtii*, the script was:

```
python3 remove_dup_name.py inputfile outputfile
```

`remove_dup_name.py` contained following code:

```
import sys
from collections import defaultdict
from Bio import SeqIO
ifname = sys.argv[1]   # xxx.fasta or  xxx.fastq
ofname = sys.argv[2]
names = defaultdict(int)
with open(ofname, "w") as ofile:
    for i, rec in enumerate(SeqIO.parse(ifname, ifname[-5:])):
        names[rec.id] += 1
        if names[rec.id] == 1:
            SeqIO.write(rec, ofile, ofname[-5:])
```

wtdbg2 pipeline was ran as

```
echo Start: $(date "+%Y-%m-%d %H:%M:%S")
wtdbg2.pl -t $threads -x ont -g $genomeSize -o $genomeName input.fastq
echo End: $(date "+%Y-%m-%d %H:%M:%S")
```

Smartdenovo pipeline was ran as:

```
awk 'NR%4==1||NR%4==2' all.fastq | sed 's/^@/>/g' > reads.fa
echo Start: $(date "+%Y-%m-%d %H:%M:%S")
smartdenovo.pl -p $genomeName -t 32 -c 1 reads.fa > dro_smart.mak
make -f dro_smart.mak
echo End: $(date "+%Y-%m-%d %H:%M:%S")
```

miniasm pipeline was ran as:

```
echo Start: $(date "+%Y-%m-%d %H:%M:%S")
minimap2 -x ava-ont -t32 all.fastq all.fastq | gzip -1 > reads.paf.gz
miniasm -f all.fastq reads.paf.gz > $genomeName.gfa
```

```
awk '/^S/{print ">"$2"\n"$3}' $genomeName.gfa | seqkit seq > $genomeName.fasta
echo End: $(date "+%Y-%m-%d %H:%M:%S")
```

NECAT pipeline first generated configuration file (necat_cfg.txt), as shown below:

```
PROJECT=$genomeName
THREADS=$threads
ONT_READ_LIST=read_list.txt
GENOME_SIZE=$genomeSize
MIN_READ_LENGTH=3000
PREP_OUTPUT_COVERAGE=40
OVLP_FAST_OPTIONS="-n 500 -z 20 -b 2000 -e 0.5 -j 0 -u 1 -a 1000"
OVLP_SENSITIVE_OPTIONS="-n 500 -z 10 -e 0.5 -j 0 -u 1 -a 1000"
CNS_FAST_OPTIONS="-a 2000 -x 4 -y 12 -l 1000 -e 0.5 -p 0.8 -u 0"
CNS_SENSITIVE_OPTIONS="-a 2000 -x 4 -y 12 -l 1000 -e 0.5 -p 0.8 -u 0"
TRIM_OVLP_OPTIONS="-n 100 -z 10 -b 2000 -e 0.5 -j 1 -u 1 -a 400"
ASM_OVLP_OPTIONS="-n 100 -z 10 -b 2000 -e 0.5 -j 1 -u 0 -a 400"
NUM_ITER=2
CLEANUP=1
USE_GRID=false
GRID_NODE=0
SMALL_MEMORY=0
CNS_OUTPUT_COVERAGE=30
FSA_OL_FILTER_OPTIONS=""
FSA_ASSEMBLE_OPTIONS=""
FSA_CTG_BRIDGE_OPTIONS=""
POLISH_CONTIGS=true
```

Then, it was run as:

```
echo Start: $(date "+%Y-%m-%d %H:%M:%S")
necat.pl bridge necat_cfg.txt
echo End: $(date "+%Y-%m-%d %H:%M:%S")
```

The read_list.txt contained the path of corresponding sequencing data; *$genomeName* was set to *E. coli, S. cerevisiae, A. thaliana*, *C. reinhardtii*, *D. melanogaster*, *O. sativa, S. pennellii* and *H. sapiens*, respectively, and *$genomeSize* was set to 4,800,000, 13,000,000, 130,000,000, 130,000,000, 120,000,000 400,000,000, 1,000,000,000 and 3,000,000,000, respectively.

For large genomes, NECAT used more corrected reads to obtain more robust assemblies. Therefore, we adjusted the parameters for *O. sativa* as shown below:

```
CNS_OUTPUT_COVERAGE=40
FSA_OL_FILTER_OPTIONS="—min_coverage 3"
```

And we adjusted the parameters for *S. pennellii* as shown below:

```
CNS_OUTPUT_COVERAGE=40
```

We also adjusted the parameters for *H. sapiens(rel3,4)* and WERI as shown below:

```
MIN_READ_LENGTH=500
PREP_OUTPUT_COVERAGE=
OVLP_FAST_OPTIONS="-n 200 -z 10 -b 2000 -e 0.5 -j 0 -u 1 -a 400"
OVLP_SENSITIVE_OPTIONS="-n 200 -z 10 -e 0.5 -j 0 -u 1 -a 400"
CNS_FAST_OPTIONS="-a 400 -x 4 -y 12 -l 500 -e 0.5 -p 0.8 -u 0"
CNS_SENSITIVE_OPTIONS="-a 400 -x 4 -y 12 -l 500 -e 0.5 -p 0.8 -u 0"
CNS_OUTPUT_COVERAGE=45
```

We also adjusted the parameters for *H. sapiens(rel6)* as shown below:

```
CNS_OUTPUT_COVERAGE=40
FSA_ASSEMBLE_OPTIONS="—max_spur_length 200000"
```

NECAT+Canu pipeline was run as

```
echo Start: $(date "+%Y-%m-%d %H:%M:%S")
canu -p $genomeName -d $genomeName genomeSize=$genomeSize maxMemory=1000
maxThreads=$threads useGrid=false -nanopore-corrected
$correctedByNECAT.fasta
echo End: $(date "+%Y-%m-%d %H:%M:%S")
```

where the parameters are the same as they in Canu pipeline. *$*`correctedByNECAT`*.fasta* was set to corrected reads generated by NECAT.

NECAT+Smartdenovo pipeline was similar to the pipeline Canu+Smartdenvo, where the input files were changed to the corrected reads generated by NECAT; the script was as follows:

```
echo Start: $(date "+%Y-%m-%d %H:%M:%S")
smartdenovo.pl -p $genomeName -t $threads -c 1 $correctedByNECAT.fasta >
$genomeName.mak
make -f $genomeName.mak
echo End: $(date "+%Y-%m-%d %H:%M:%S")
```

Canu+Flye and NECAT+Flye pipelines were similar to Flye pipeline, where the `--nano-raw` was changed to `--nano-corr` and using corrected reads generated by Canu or NECAT as input files; the script was as follows:

```
echo Start: $(date "+%Y-%m-%d %H:%M:%S")
flye --nano-corr correctedByNECATorCanu.fasta --out-dir $genomeName --
genome-size $genomeSize --threads $threads
```

```
echo End: $(date "+%Y-%m-%d %H:%M:%S")
```

Canu+NECAT pipeline was run as:

```
echo Start: $(date "+%Y-%m-%d %H:%M:%S")
necat.pl bridge necat_cfg.txt
echo End: $(date "+%Y-%m-%d %H:%M:%S")
```

where the *necat_cfg.txt* is similar to the one used by NECAT pipeline. But we removed the parameter `ONT_READ_LIST` and add the parameter `CNS_READ_LIST` as follow:

```
CNS_READ_LIST=read_list.txt
```

The *read_list.txt* contained the path of corrected reads by Canu.

Supplementary Note 11: Validation of the WERI genome

The new WERI assembly from Nanopore data was polished four times using the same scripts as those shown in Supplementary Note 13. We compared the WERI assembly against human reference genome hg38. The newly-assembled genome was aligned to the reference genome, and the Mummer plot between them was generated using MUMmer (v4.0)[12] with the following script (Supplementary Figure 2):

```
nucmer --mum -l 10 -c 1000 --banded  ${ref.fasta} ~/project/weri/ONT_asm.fasta
dnadiff -d out.delta
mummerplot out.delta --fat -f -png
```

Because MUMmer was operated using a unique anchor matching option to accelerate the alignment, some repetitive sequences remained unaligned. The entire process of alignment and figure generation can be reproduced using scripts available on the MHAP home page[24] (assuming that Perl, Python, and MUMmer[12] are placed in the correct path), and by running the script below; this generates a figure designated as asm.pdf (Figure 3).

```
sh makeHuman.sh ref.fasta asm.fasta
```

Based on out.rdiff file output by dnadiff, structural differences (>10 bp) were extracted using the following scripts:

```
awk ' {if($3<=$4&&$7*$7>100) print $1 "\t" $3"\t"$4"\t"$2"\t"$7
if($3>$4&&$7*$7>100)  print  $1  "\t"  $4"\t"$3"\t"$2"\t"$7  }'  ./out.rdiff  >
weri_10.bed
wc -l weri_10.bed
```

We then used a custom script to convert the SV regions in the WERI assembly genome to the reference hg38:

```
awk '{if($3>$4&&$7*$7>100) print $1"\t"$4"\t"$3"\t"$2"\t"$7"\t"($3-
1)"\t"($4+1)
if ($3<=$4&&$7*$7>100) print $1"\t"$3"\t"$4"\t"$2"\t"$7"\t"($3-
1)"\t"($4+1)}' ./out.qdiff > hg38_gap.tsv
awk '{if($3>$4&&$7*$7>100) print $1"\t"$4"\t"$3"\t"$2"\t"$7"\t"($3-
1)"\t"($4+1)
if ($3<=$4&&$7*$7>100) print $1"\t"$3"\t"$4"\t"$2"\t"$7"\t"($3-
1)"\t"($4+1)}'  ./out.rdiff > weri_gap.tsv
python3 query.py  -c hg38_gap.tsv -w weri_gap.tsv -a out.1coords >
```

```
weri2hg38.tsv
```

To validate SV regions detected in WERI, we re-aligned the original sequencing data with SV regions±1000 bp. SV regions were extracted with:

```
awk '{if($5>=$6) print $4,":",$6-1000,"-",$5+1000
if($5<$6) print $4,":",$5-1000,"-",$6+1000}' weri2hg38.tsv | sed 's/ //g' >
qgap
for i in $(cat qgap);do samtools faidx ./ref.fasta $i >> all_gap.fasta;done
# re-align the raw nanopore reads to all_gap.fasta
minimap2 -x map-ont -t $NPROC ./all_gap.fasta ./fq > all_gap.paf
```

Then, we calculated the number of SV regions with read coverage:

```
awk      '($8<=($7-1000))&&($9>=1000){print      $6,"\t",$7,"\t",$8,"\t",$9}'
all_gap.paf > real_map
awk '{print $1}' real_map | sort | uniq -c | tee real_map_list | wc l
awk '{split($2,a,"\[:-\]");print a[1],"\t",(a[2]+1000),(a[3]-
1000),$1}' ./real_map_list > real_map_list_raw
#generate merge.tsv
cat ./real_map_list_raw | xargs -n 4 -P 10 ./merge.sh
# generate merge.bed
awk '{if($2>$3){print $1,$3,$2,$4}else{print $1,$2,$3,$4}}' ./merge.tsv |
sed 's/ /\t/g'  | grep -v 'chrY' > merge.bed
```

We also aligned the raw nanopore long reads and Illumina short reads to human reference genome hg38, and used Sniffles[32] and Lumpy_sv[33] to call SVs in mapping results using the scripts shown below:

Sniffles:

```
export PATH=/ /software/Sniffles-1.0.10/bin/sniffles-core-1.0.10:$PATH
export PATH=/ /software/ngmlr-0.2.7:$PATH
ngmlr -t $NPROC -r $refsequence -q $fq -o reads.sam -x ont
samtools view -bS reads.sam | samtools sort -@ $NPROC - -o reads.sorted.bam
sniffles -t $NPROC -m reads.sorted.bam -v tgs.weri.vcf
```

Lumpy_sv:

```
bwa  mem  -R  "@RG\tID:id\tSM:sample\tLB:lib"  reference.fasta  sample.1.fq
sample.2.fq |  samblaster  --excludeDups  --addMateTags  --maxSplitCount  2  --
minNonOverlap 20 | samtools view -S -b -  >  sample.bam
samtools view -b -F 1294 sample.bam
| samtools sort  -o sample.discordants.sorted.bam
samtools view -h sample.bam \
  | scripts/extractSplitReads_BwaMem -i stdin \
  | samtools view -Sb - \
```

```
 | samtools sort  -o sample.splitters.sorted.bam
lumpyexpress \
    -B sample.bam \
    -S sample.splitters.bam \
    -D sample.discordants.bam \
    -o output.vcf
export PATH= /software/VCFtools/bin:$PATH
cat ngs.weri.vcf | vcf-sort > sorted.ngs.vcf
cat tgs.weri.vcf | vcf-sort > sorted.tgs.vcf
bzip sorted.ngs.vcf
bzip sorted.tgs.vcf
bcftools stats ./sorted.ngs.vcf.gz > ngs.stat
bcftools stats ./sorted.tgs.vcf.gz > tgs.stat
#index
tabix -p vcf sorted.ngs.vcf.gz
tabix -p vcf sorted.tgs.vcf.gz
# generate 0000.vcf  0001.vcf  0002.vcf  0003.vcf
-l
# weri SV and ngs
bedtools intersect -a ./merge.bed -b ./sorted.ngs.bed -wa -loj | awk
'$5!="."{print}' | wc -l
# weri SV, ngs and tgs overlap
bedtools intersect -a ./merge.bed -b ./comm.ngs2tgs.bed -wa | wc -l
bcftools isec sorted.ngs.vcf.gz sorted.tgs.vcf.gz -p ./
# convert vcf to bed
awk '{split($8,a,"RE=");print $1,$2,($2+1),a[2]}' ./sorted.tgs.vcf | grep -v
'#' | grep -v 'chrY' | sed 's/ /\t/g'> sorted.tgs.bed

awk '{split($10,a,":");print $1,$2,($2+1),a[2]}' ./sorted.ngs.vcf | grep -v
'#' | grep -v 'chrY' | sed 's/ /\t/g' > sorted.ngs.bed
echo "CHROM POS ID REF ALT QUAL FILTER Coverage" > 0002.head
awk '{split($10,a,":");print $1,$2,$3,$4,$5,$6,$7,a[2]}' ./0002.vcf | grep -v
'#' | cat 0002.head - > ngs.commom.add_cov.vcf
grep -v CHROM ./ngs.commom.add_cov.vcf | awk '{print $1,$2,($2+1)}' | sed 's/
/\t/g' > comm.ngs2tgs.bed
# weri SV and tgs
bedtools intersect -a ./merge.bed -b ./sorted.tgs.bed -wa -loj | awk
'$5!="."{print}' | wc -l
```

Supplementary Note 12: Overlap-filtering strategy

Overlap-filtering is critical in genome assembly. High-error-rate overlaps introduce errors and complicate assembly. Conversely, an overly strict filtering strategy can reduce contiguity of the results. Error distribution of sequencing data varies greatly. In order to adapt to different data, we adopted a heuristic filtering strategy to remove high-error-rate overlaps. Two metrics, the identity obtained by dividing length of the overlap by the number of matching bases, and the overhang that is the distance of an overlap from the 5' or 3' end of the read, are used to identify high error rate overlaps.

First, we examined overlap identities. For each read, we collect its overlaps and compute the mean of identities of the overlaps as its identity. After obtaining all read identities, we computed the weighted median (m_g^{id}) and weighted median absolute deviation (MAD_g^{id}) of them, where the weight is the read length. We used the following formula to calculate global threshold of overlap identity (th_g^{id}):

$$th_g^{id} = \min(m, m_g^{id}) - n * k * MAD_g^{id}. \quad (1)$$

Here k is equal to 1.4862, a constant scale factor multiplied by *MAD* to obtain an estimation of the standard deviation σ. According to our experience, m and are n are set to 0.98 and 6, respectively. After obtaining global threshold for overlap identity, we calculated the local threshold. For each read, we accumulate the lengths of its overlaps. If the sum was less than $\max(c_{min}, 0.5 * c) * l$, where c_{min} is a user-set parameter (default value is 25), c is the coverage of corrected reads, and l is read length, we set the local threshold th_l^{id} to global threshold th_g^{id}, because the data were too small to show statistical significance. Otherwise, we sorted the overlaps in descending order according to the product of overlap identity and overlap length. We collected the first several overlaps in which the sum of their lengths was no more than $\max(2 * c_{min}, 1.5 * c) * l$. Then, we computed weighted median (m_l^{id}) and weighted median absolute deviation (MAD_l^{id}) of these overlaps, where the weight was overlap length. Local identity threshold th_l^{id} is was set to $\max\big(th_g^{id}, \big(\min(m, m_l^{id}) - n * k *$

$MAD_l^{id}\big)\big)$, where *m* and *n* were set to 0.99 and 6 by default. Next, we used th_l^{id} to filter out the read overlaps. If overlap identity was less than th_l^{id}, the overlap was removed.

We used a similar process to assess read overhang in the overlaps. For each read, we collected the maximum of its overhangs. Then, we computed the weighted median (m_g^{oh}) and weighted median absolute deviation (MAD_g^{oh}), where weight was read length. The formula used to calculate global threshold of an overhang is provided in (2), where *m* and *n* were set to 30 and 6 by default, respectively.

$$th_g^{oh} = \max\big(m, m_g^{oh}\big) + n * k * MAD_g^{oh}. \tag{2}$$

Next, we collected read overhangs at the 5' or 3' end separately, and computed the local thresholds for them. For each end, if the number of read overhangs is less than $max(c_{min}, 0.5 * c)$, then c_{min} is a user-set parameter (default value is 25), *c* is coverage of corrected reads, and local threshold th_l^{oh} is set to global threshold th_g^{oh}. Otherwise we sorted overhangs in ascending order according to results obtained by dividing the overlap overhang by overlap length. We collected the first several overhangs, the number of whom is no more than $\max(2 * c_{min}, 1.5 * c)$. Then, we computed weighted median (m_l^{oh}) and weighted median absolute deviation (MAD_l^{oh}) for these overhangs, where weight was overlap length. Local identity threshold th_l^{oh} was set to $\min\big(th_g^{oh}, \big(\max\big(m, m_l^{oh}\big) + n * k * MAD_l^{oh}\big)\big)$, where *m* and *n* were set to 10 and 6 by default, respectively. The overlap was removed if the read overhang at 5' or 3' end was greater than th_l^{oh} of the corresponding end.

In addition to assessing overlap identity and read overhang, we used the following filtering strategies.

1. We calculated the coverage for each base in the reads according to overlaps between them. For each read, we obtained three metrics, minimum coverage of all bases (c_{min}), maximum coverage of all bases (c_{max}), and the difference between minimum

coverage and maximum coverage (c_{diff}). The procedure used three thresholds, designated as *min_coverage, max_coverage*, and *max_diff_coverage*, to assess read metrics and automatically select thresholds based on statistical results. If c_{min} is less than *min_coverage*, c_{max} is larger than *max_coverage*, and c_{diff} is larger than *max_diff_*coverage, the reads and related overlaps are removed. Our analysis of the yeast dataset indicated that *min_coverage* should be set to the first value not exceeding 30% of the value for the first trough of the histogram of all c_{min}s, as shown in Supplementary Figure 15. We suggest that *max_coverage* and *max_diff_coverage* be set to (100-*x*)-th percentile (*x*=0.01 by default). These thresholds can also be specified by users. After some of the reads are filtered out, coverages of each read may change. This filtering strategy is executed twice to increase robustness of the results.

2. In this step, we assessed the overlaps and counted the number of reads having an overlap with the first read and covering the 5'- or 3'-end of the second read. If the number was less than *min_coverage* - 1, this overlap was filtered out.
3. The contained reads and related overlaps were filtered out.
4. Finally, for each read, we sorted the overlaps covering its 5'- or 3'-end by aligned length, respectively. The best overlaps can be selected using *bestn*, a parameter specified by users.

Supplementary Note 13: Genome polishing and assembly validation

Different polishing strategies were used for different genome-assembly pipelines (NECAT, Canu[28], and Canu+smartdenovo[29]) and different species (*E. coli, S. cerevisiae, A. thaliana*, *D. melanogaster*, *C. reinhardtii*, *O. sativa* and *S. pennellii*):

1. Nanopolish (v0.10.2)[8] was used to further polish the genome using fast5 files and corresponding fasta/fastq files. Finally, the genome was polished three times using NGS data with Pilon (v1.22)[9] and generated the final genome.

2. For the *A. thaliana*, we used the Arrow in smrtlink (v5.1.0)[10] to polish the draft genome with Sequel Bam files because the raw fast5 files required by Nanopolish were not available.

3. For the *O. sativa* and Human, we used minimap2[27] (v2.10-r761) with "-x map-ont" and Racon[11] (v1.3.1) with default parameters to polish the draft genome four times using raw reads.

4. For *S. pennellii*[34], the assemblies were polished five times using NGS data with Pilon (v1.22).

We used QUAST[6] (5.0.2) to evaluate the matrics number of contigs, NG50, NGA50, number of misassemblies and QV($\log_{10}(\frac{100kbp}{\#\ mismatches\ per\ 100\ kbp + \#\ indels\ per\ 100\ kbp})$) of assemblies. QUAST was run using the "--min-contig 5000 --min-identity 90" options for *E. coli*, using the "--min-contig 5000 --large --min-identity 90" options for *S. cerevisiae, A. thaliana* and *O. sativa*, using the "--min-contig 5000 --large --min-identity 90 --fragmented" options for *D. melanogaster, S. pennellii*, and using the "--min-contig 50000 --large --min-identity 90 --fragmented" options for Human.

BUSCO[35] (4.0.6) was run to evaluate gene completeness of assemblies for all species. We used the following script:

```
busco -i $contigs -m geno  -l $lib -e 0.001 --offline -o output
```

where $contigs was set to one of assemblies and $lib was set to corresponding OrthoDB

v10 dataset. We used datasets enterobacterales, saccharomycetes, brassicales, diptera, chlorophyta, poales, solanales and primates for *E. coli*, *S. cerevisiae*, *A. thaliana*, *D. melanogaster*, *C. reinhardtii*, *O. sativa*, *S. pennellii* and *H. sapiens*, respectively. Those datasets can be downloaded from https://busco.ezlab.org/busco_v4_data.html.

Alignments and validation results of statistical analysis are shown in Supplementary Figures 6-13.

We then mapped the assembled genomes onto their reference genomes, and counted single-nucleotide polymorphisms (SNPs) and large indels using dnadiff[36] and GEGE[37]. The five genome assemblies for *E. coli*, *S. cerevisiae*, *A. thaliana*, *D. melanogaster* and *C. reinhardtii* and were aligned to their reference genomes and plotted using MUMmer (v4.0)[12]. Results were generated using the following scripts:

```
nucmer --mumreference -l 100 -c 1000 -d 10 --banded -D 5 ${ref.fasta}
${asm.fasta}
delta-filter -i 95 -o 95 out.delta> out.best.delta
dnadiff -d out.best.delta
mummerplotout.best.delta --fat -f -png
```

We also compared genome assemblies for *E. coli, S. cerevisiae, A. thaliana, C. reinhardtii*, and *D. melanogaster* generated using Canu, Canu+Smartdenovo, Smartdenovo, miniasm, wtdbg2, Flye, and NECAT pipelines. The following scripts were used to evaluate Indel gaps in these genome assemblies:

```
awk'{if($2=="GAP"&&sqrt($7*$7)>=10) {print $0 }}' ${out.qdiff} >
indelM10.txt
awk'{if($2=="GAP"&&sqrt($7*$7)<10) {print $0 }}' ${out.qdiff} > indelL10.txt
```

SNPs and indels between the assembly genome and reference genome are listed in Supplementary Table 6.

Supplementary Note 14: Analysis of repeat regions in *D. melanogaster*

Repeat regions are one of the greatest challenges in genome assembly. To assess transposable_element (TE)[17] resolution in NECAT assembly, we analyzed the TE repeat families and aligned the annotated *D. melanogaster* genome[16] to the seven assembled contigs from the genome assemblies pipelines (Canu, canu+smartdenovo, Smartdenovo, miniasm, wtdbg2, Flye, and NECAT). Genome FlyBase 5.57_FB2014_0310 was downloaded from:

ftp://ftp.flybase.net/genomes/dmel/dmel_r5.57_FB2014_03/fasta/dmel-all-gene-r5.57.fasta.gz.

The annotated gff file was downloaded from:

ftp://ftp.flybase.net/genomes/dmel/dmel_r5.57_FB2014_03/gff/dmel-all-r5.57.gff.gz.

Transposable element (TE) features were extracted and converted to bed file using:

```
awk'$2=="FlyBase"&&$3=="transposable_element" {print $0}' <dmel-all-
r5.57.gff> > <TE.gtf>
awk'{print $1"\t"$4"\t"$5"\t"$3"_"NR}' <TE.gtf> > <TE.bed>
```

Then, the pipeline was executed as:

```
assembled_feature_pipeline.sh -a <asm.fasta> -r <reference.fasta> -f
<TE.bed>
```

After running the scripts, the final output file, called results/FINAL.REPORT, was generated and used to identify TE with ⩾100% Pct_length and corresponding Pct_ident. The repeat familes, *roo* and *juan*, were extracted from the FINAL.REPORT file. The annotated region from *roo* and *juan* families can be extracted from dmel-all-r5.57.gff. The results are shown in Supplementary Table 7.

Supplementary Note 15: Analysis of telomere assembly

LRs provide considerable advantages in reconstructing the repetitive heterochromatic regions of eukaryotic chromosomes. Telomeres play important roles in chromosome replication of all eukaryotic genomes. Nanopore LR sequencing presents distinct advantages in telomere assembly. To validate the effectiveness of using Nanopore data, we evaluated long-read sequencing in reconstruction of heterochromatic sequences in telomeric regions of *S. cerevisiae*.

The *S. cerevisiae* S288C other features database was downloaded from http://downloads.yeastgenome.org/sequence/S288C_reference/other_features/other_features_genomic.fasta.gz. We mapped selected *S. cerevisiae* telomeric repeats to *S. cerevisiae* W303 assemblies generated using Canu, Canu+Smartdenovo, Smartdenove, miniasm, wtdbg2, Flye and NECAT.

The features were aligned to the assembly using the following scripts:

```
nucmer --maxmatch<asm.fasta><features.fasta>
show-coords -lrcTHout.delta |sort -nk12 |awk'{if ($7> 85 && $11> 50) print $0}' | grep TEL | sort -rnk8 >tels.coords
```

The contigs containing telomeric features within 1 kbp of contig ends were then identified. The results are shown in Supplementary Table 8.

Supplementary Note 16: Validation of *H. sapiens* NA12878

To validate the performances of NECAT and Canu, each polished assembly was aligned to reference genome hg38 with MUMmer (v4.0)[12], after which tiling figures were generated (Supplementary Figure 12). The genome was polished four times using Nanopore data with Racon[11] (v1.3.1) and minimap2[27] (v2.10-r761), after which the final genome was generated using the following code:

```
minimap2 -x map-ont -t $NPROC $DRAFT reads.fastq > ONTmin_IT0.paf
time racon -m 8 -x -6 -g -8 -w 500 -t $NPROC reads.fastq ONTmin_IT0.paf $DRAFT > ONTmin_IT1.fasta
minimap2 -x map-ont -t $NPROC ONTmin_IT1.fasta reads.fastq > ONTmin_IT1.paf
time racon -m 8 -x -6 -g -8 -w 500 -t $NPROC reads.fastq ONTmin_IT1.paf ONTmin_IT1.fasta > ONTmin_IT2.fasta
minimap2 -x map-ont -t $NPROC ONTmin_IT2.fasta reads.fastq > ONTmin_IT2.paf
time racon -m 8 -x -6 -g -8 -w 500 -t $NPROC reads.fastq ONTmin_IT2.paf ONTmin_IT2.fasta > ONTmin_IT3.fasta
minimap2 -x map-ont -t $NPROC ONTmin_IT3.fasta reads.fastq > ONTmin_IT3.paf
time racon -m 8 -x -6 -g -8 -w 500 -t $NPROC reads.fastq ONTmin_IT3.paf ONTmin_IT3.fasta > ONTmin_IT4.fasta
```

The custom scripts, used to convert the output into a format accepted by ColoredChromesomes.pl (http://sourceforge.net/projects/cchrom/), are shown below:

```
python makeMappings.py asm_refhg38.1coords 10000 > asm.tiling
perl convertToChr.pl human.chr.map asm.tiling human.lanes human.chrPos > asm.cfg
perl coloredChromosomes.pl --chromosomeSpec asm.cfg -o asm.ps
ps2pdf asm.ps
```

Because MUMmer was set to use a unique anchor matching option to accelerate the alignment, some repetitive sequences remained unaligned. To avoid displaying these regions as gaps in tiling, the conversion script chained together consecutive alignments from the same contig if alignment gap in the reference was less than 10,000 bp. Thus, breaks in the resulting tiling occurred whenever a contig switch occurred, or if there was a >10,000 bp gap between two alignments of the same contig. The entire process of alignment and figure generation can be reproduced using the scripts available on the MHAP home page[24] (assuming that Perl, Python, and MUMmer[12] are placed in the correct path), and by running the script shown below; this generates a figure designated as asm.pdf (Supplementary Figure 14).

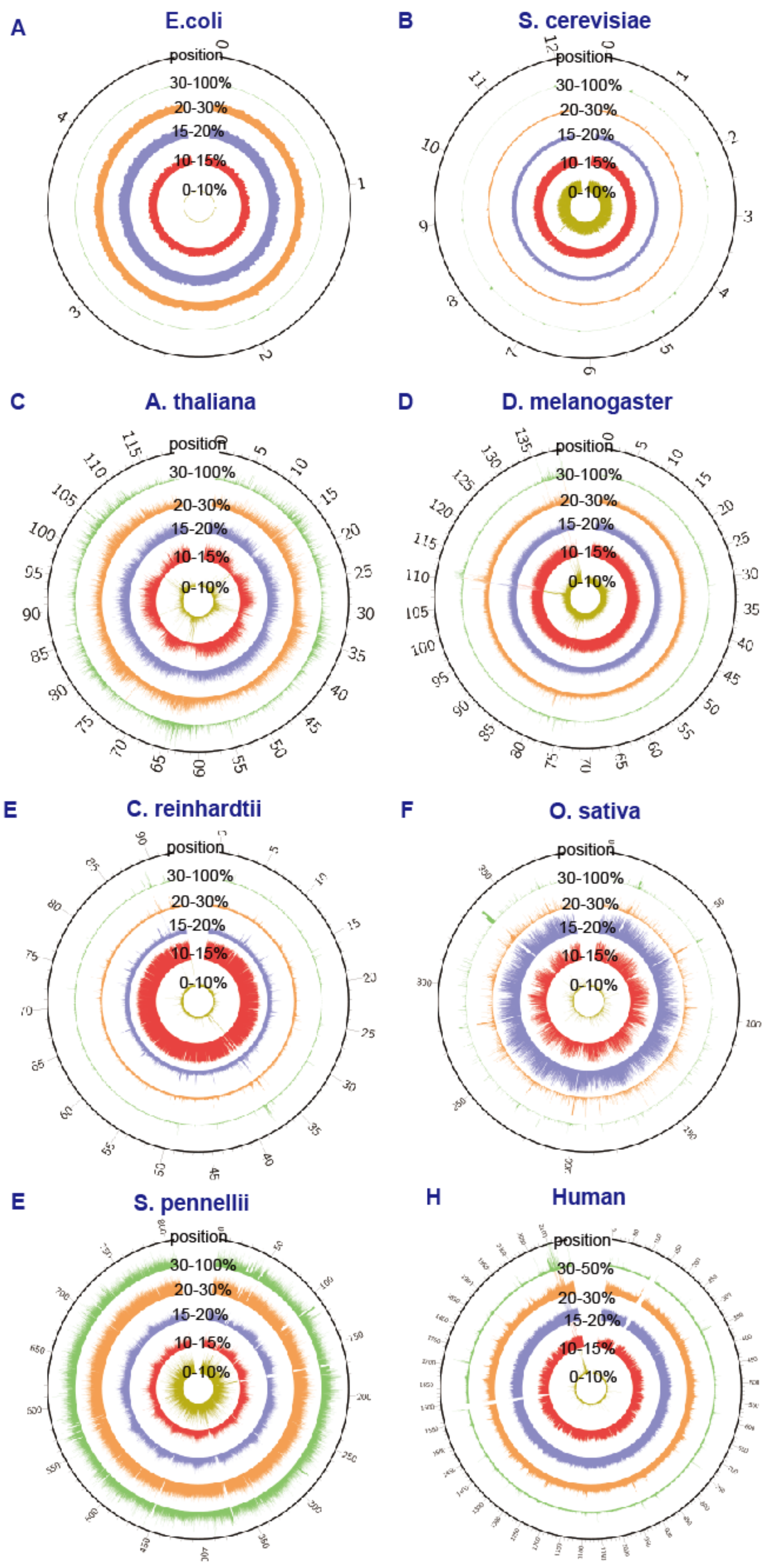

Supplementary Figure 1

Sequencing error distribution for aligned Nanopore raw long reads on different reference-genome positions in the nanopore datasets. (I: genome position; II: percentage of reads with 30-100% sequencing error rate; III: 25-30%; IV: 20-25%; V: 15-20%; VI: 10-15%; VII: 0-10%).

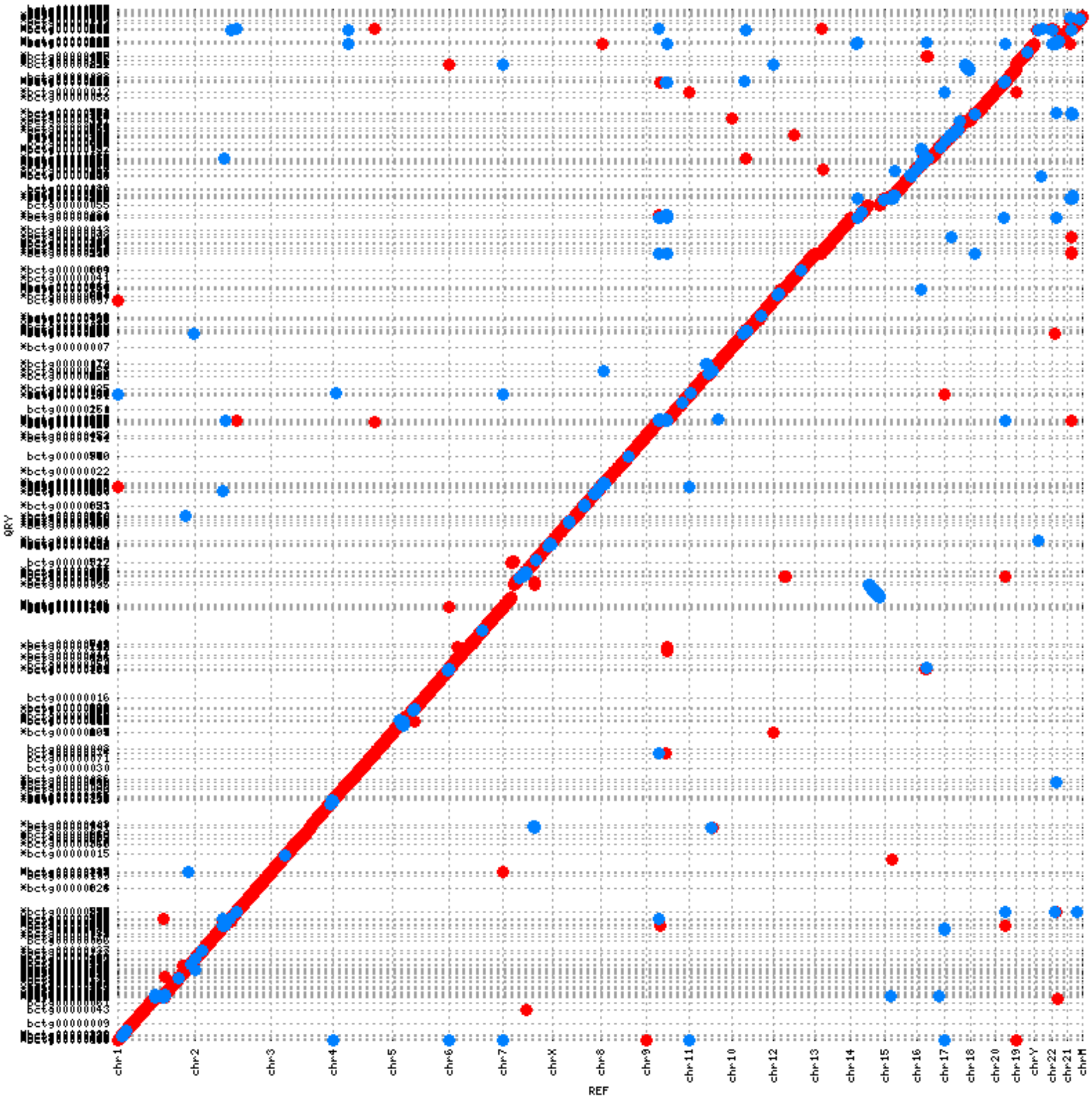

Supplementary Figure 2

Mummerplot of new assembled WERI Nanopore contigs and hg38 reference genome. An alignment dotplot shows the relationship between the contig assembled using Nanopore (y-axis) and GRCh38 reference genome (x-axis). Contig and chromosome boundaries are displayed as dotted lines (horizontal and vertical, respectively).

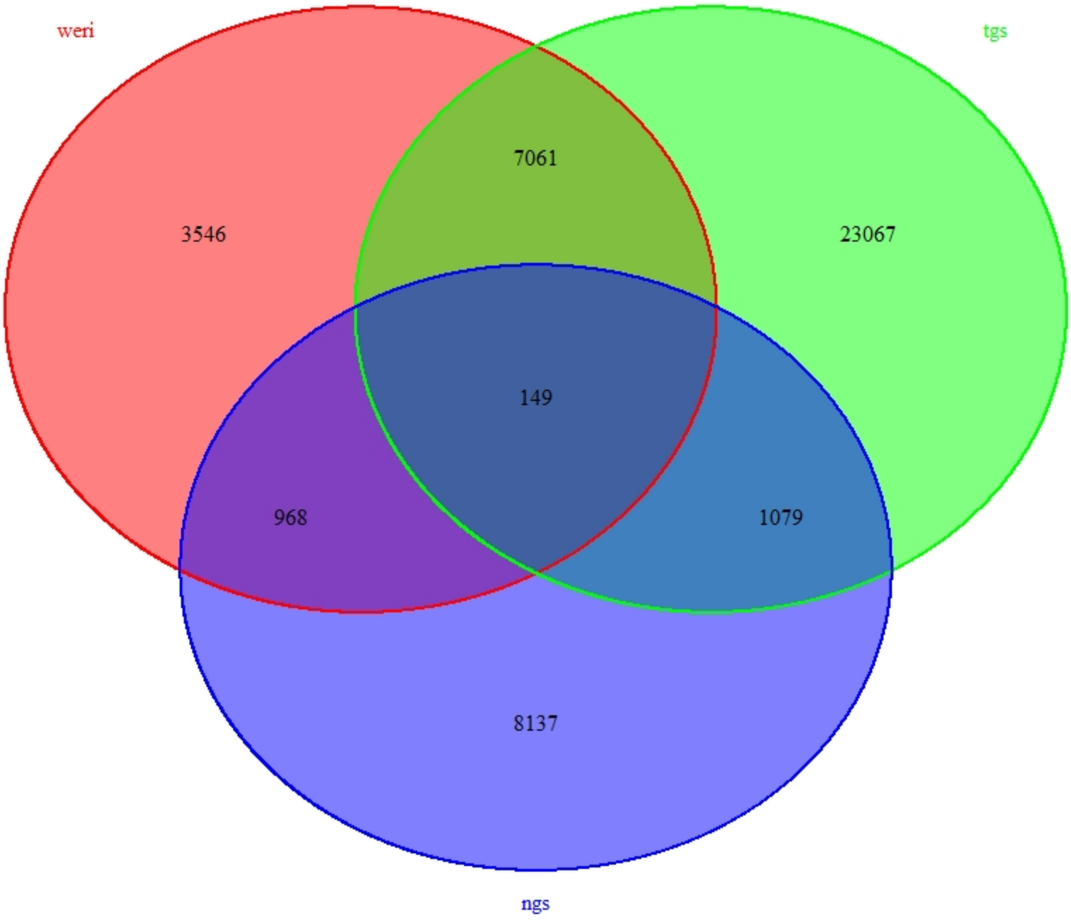

Supplementary Figure 3

The number of identified SVs detected with WERI, TGS, and NGS.

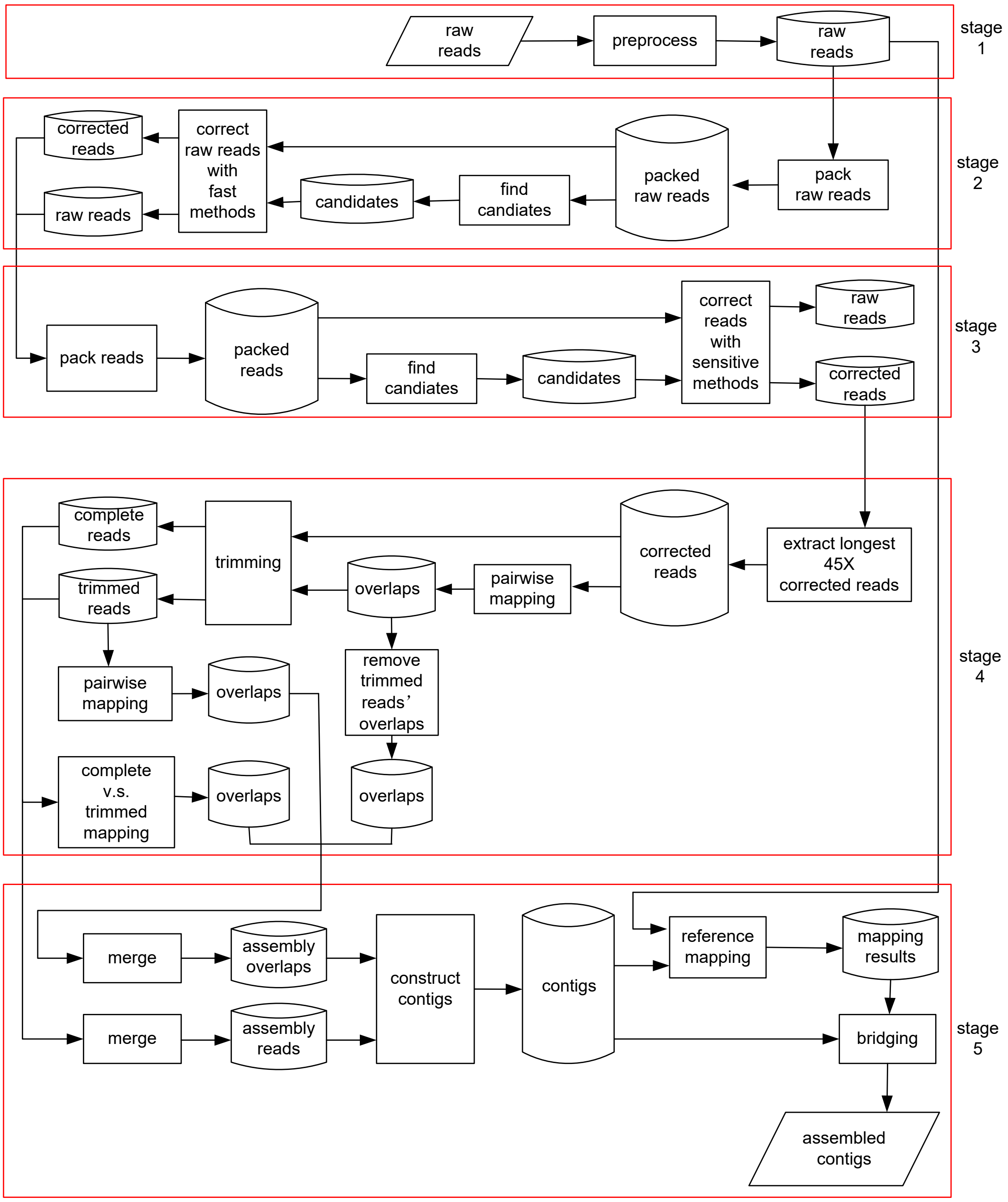

Supplementary Figure 4

NECAT architecture. Stage 1: preprocess; Stage 2: step one of correction; Stage 3: step two of correction; Stage 4: trimming; Stage 5: assembly.

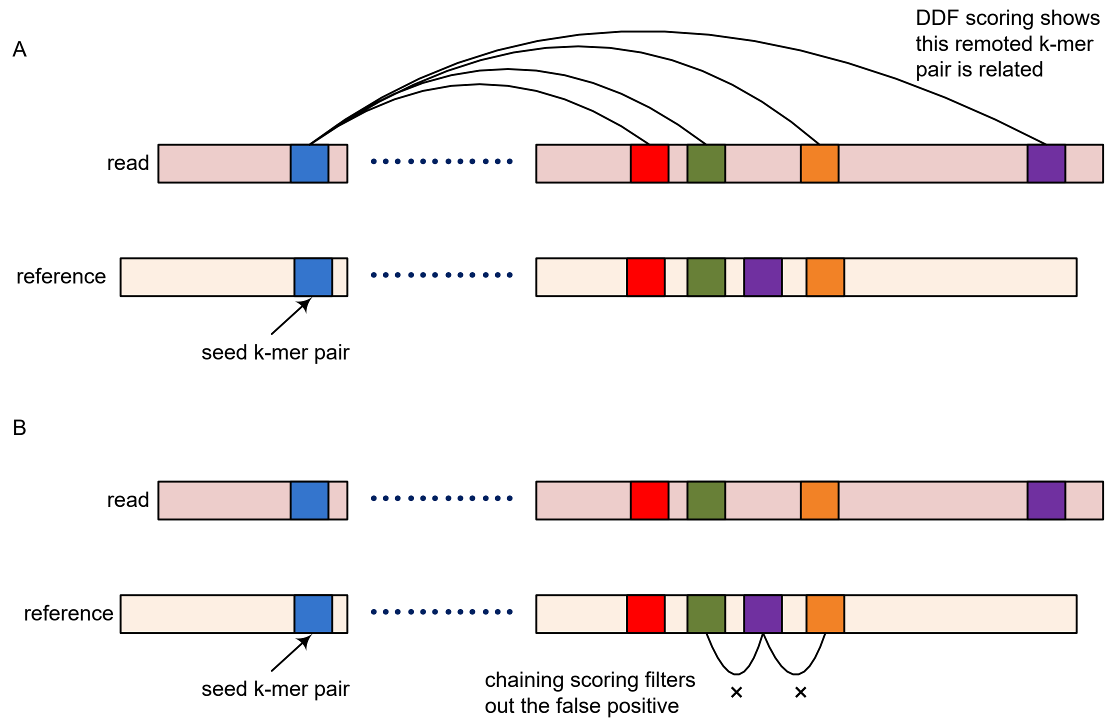

Supplementary Figure 5

Removing false positives with chaining technique. (A): The candidate k-mer pair (blue) and its four remote related k-mer pairs detected by DDF scoring. (B)Chaining is used to remove false positives (purple) by examining positions with adjacent k-mer pairs.

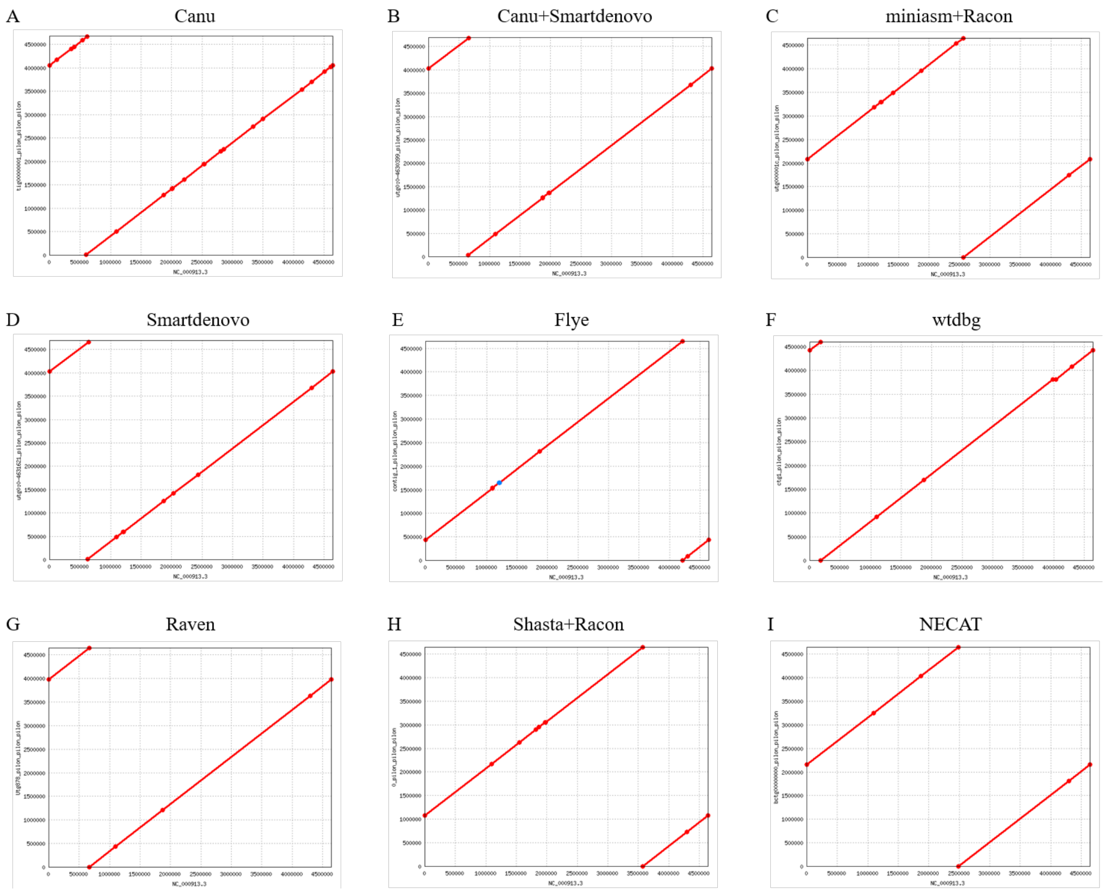

Supplementary Figure 6

Mummerplot of assembled contig and *E. coli* reference genome. An alignment dotplot shows the relationship between the assembled contig of *E. coli* K12 (y-axis) and *E. coli* K12 reference genome (x-axis). The assembled single contig was mapped onto the reference genome and covered the entire genome. The assembled contig was arbitrarily shifted because the *E. coli* chromosome is circular; this does not represent assembly error.

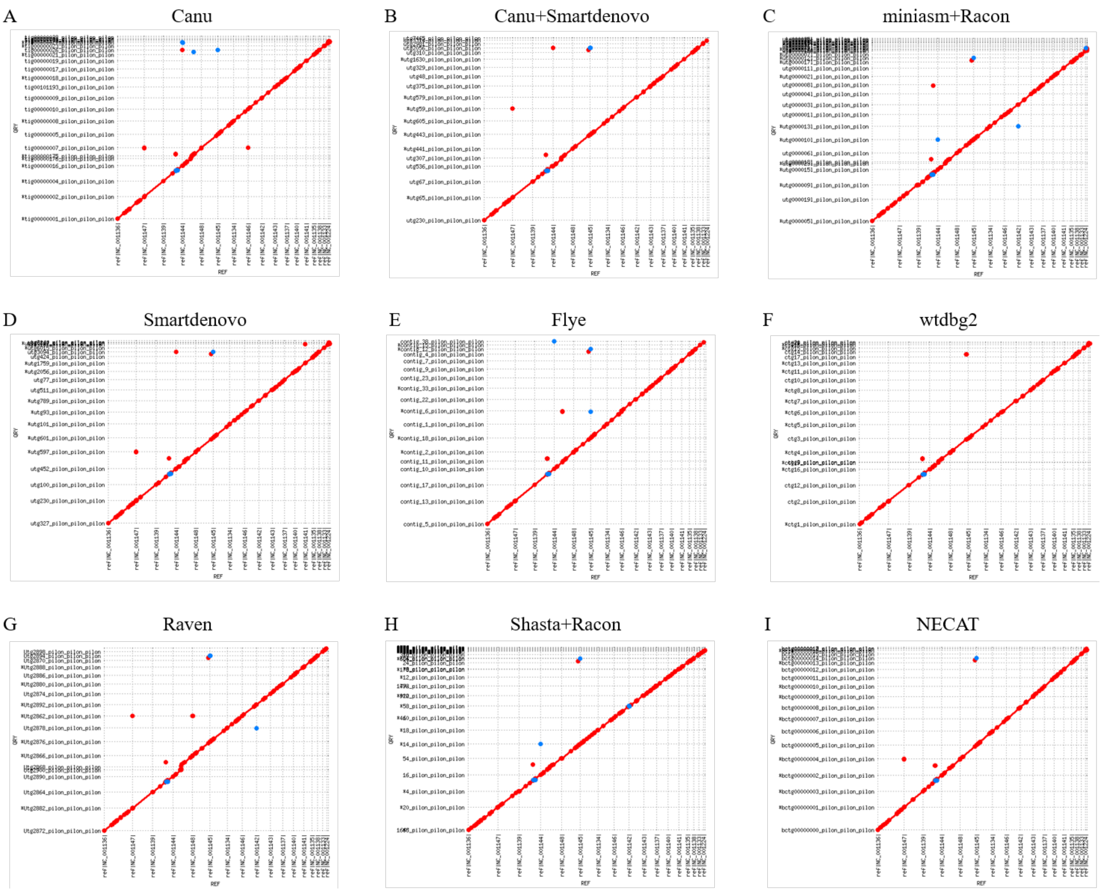

Supplementary Figure 7

Mummerplot of the assembled contig and *S. cerevisiae* reference genome. An alignment dotplot shows the relationship between the contig assembled using Nanopore (y-axis) and *S. cerevisiae* reference genome (x-axis). Contig and chromosome boundaries are displayed as dotted lines (horizontal and vertical, respectively).

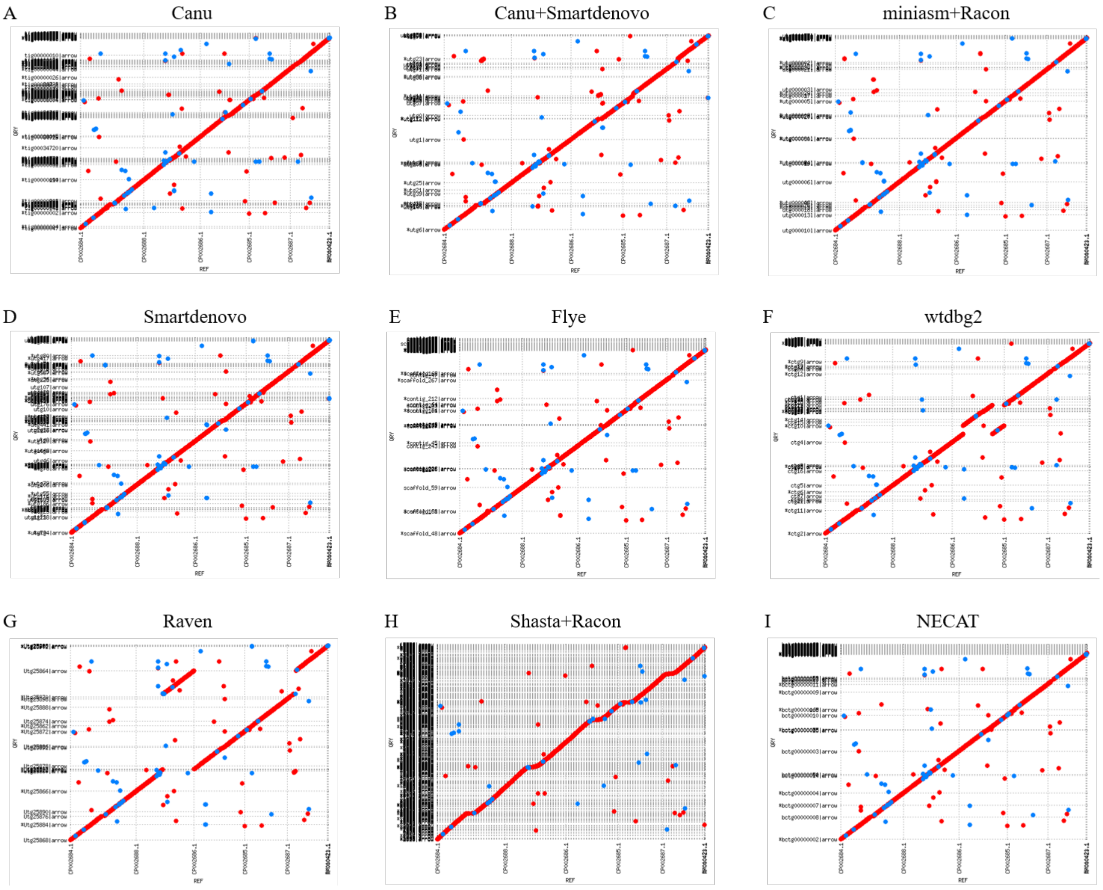

Supplementary Figure 8

Mummerplot of the assembled contig and *A. thaliana* reference genome. An alignment dotplot shows the relationship between the contig assembled using Nanopore (y-axis) and *A. thaliana* reference genome (x-axis). Contig and chromosome boundaries are displayed as dotted lines (horizontal and vertical, respectively).

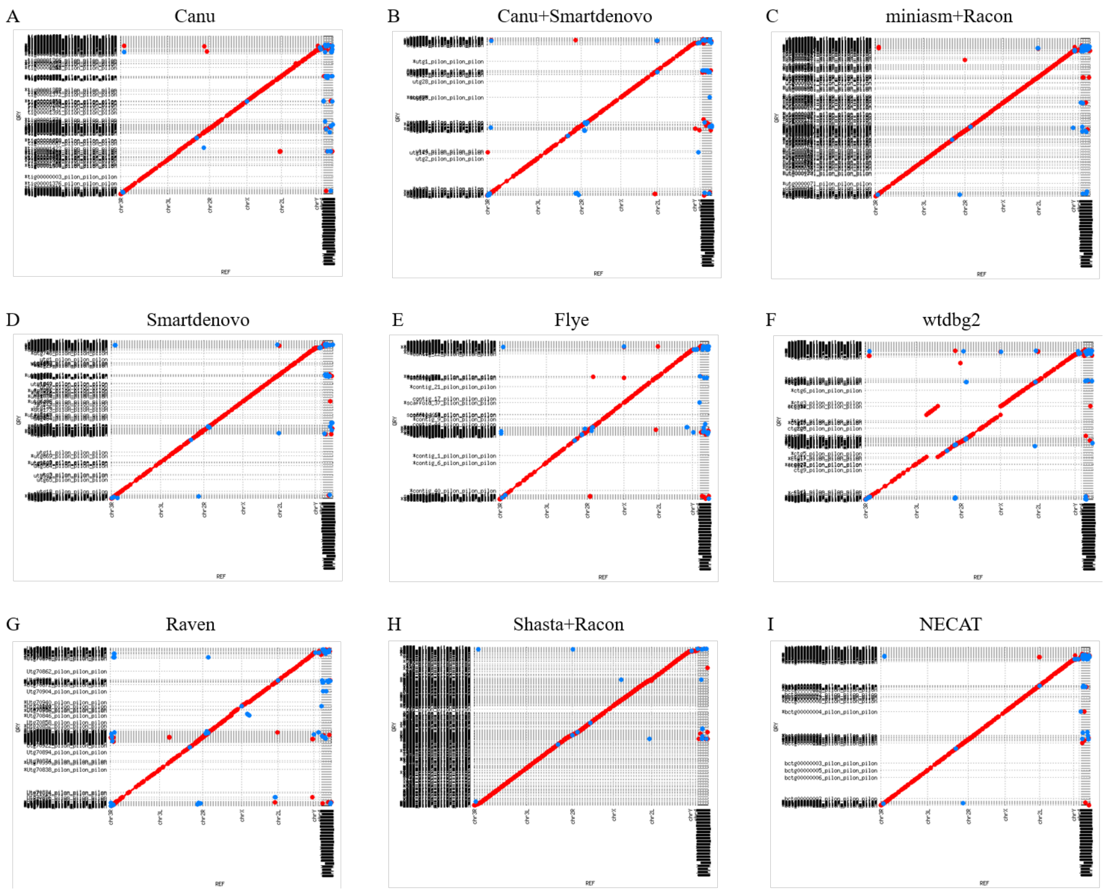

Supplementary Figure 9

Mummerplot of the assembled contig and *D. melanogaster* reference genome. An alignment dotplot shows the relationship between the contig assembled using Nanopore (y-axis) and *D. melanogaster* reference genome (x-axis). Contig and chromosome boundaries are displayed as dotted lines (horizontal and vertical, respectively).

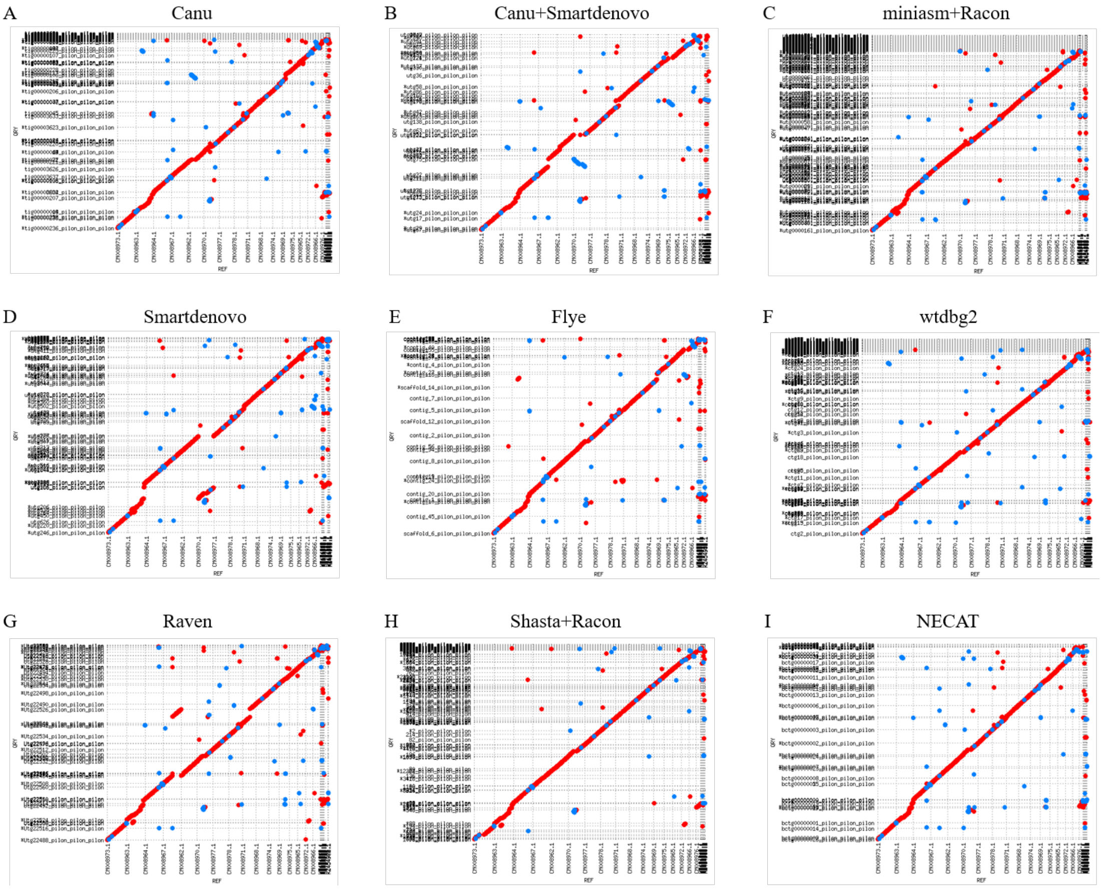

Supplementary Figure 10

Mummerplot of the assembled contig and *C. reinhardtii* reference genome. An alignment dotplot shows the relationship between the contig assembled using Nanopore (y-axis) and *C. reinhardtii* reference genome (x-axis). Contig and chromosome boundaries are displayed as dotted lines (horizontal and vertical, respectively).

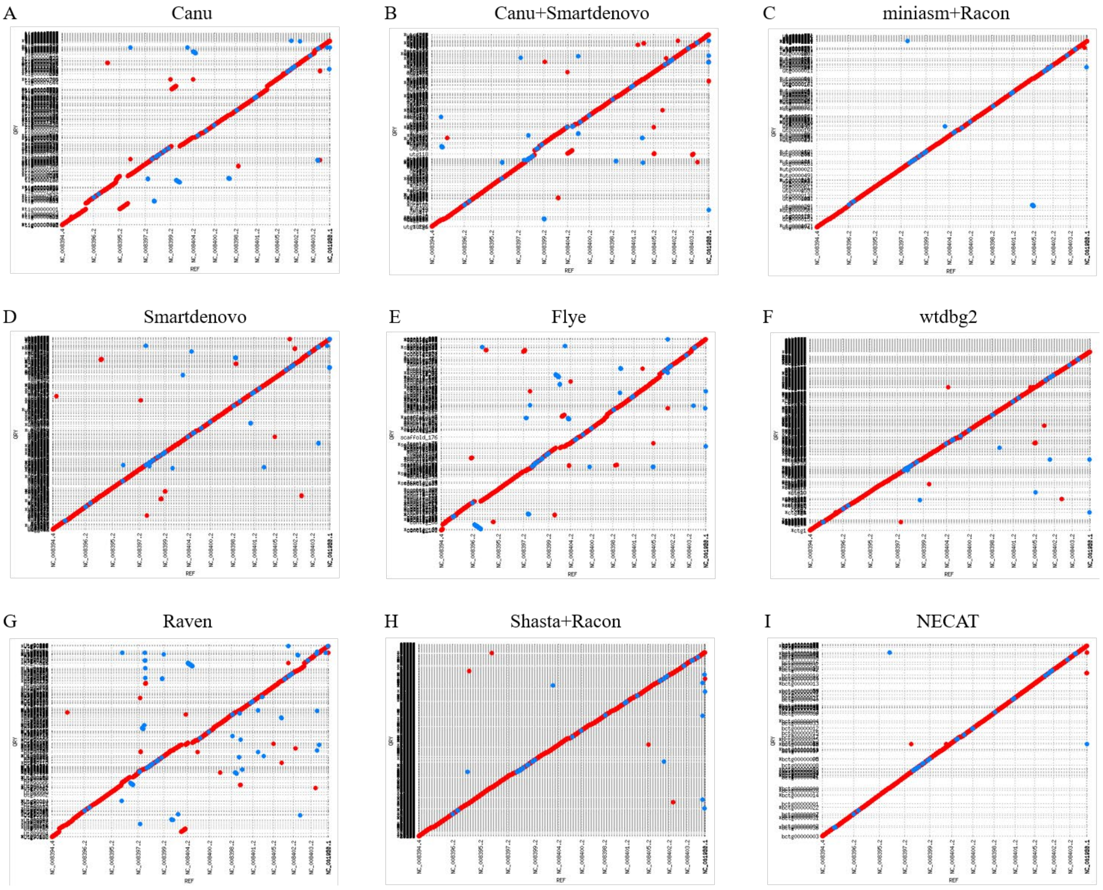

Supplementary Figure 11

Mummerplot of the assembled contig and *O. sativa* reference genome. An alignment dotplot shows the relationship between the contig assembled using Nanopore (y-axis) and *O. sativa* reference genome (x-axis). Contig and chromosome boundaries are displayed as dotted lines (horizontal and vertical, respectively).

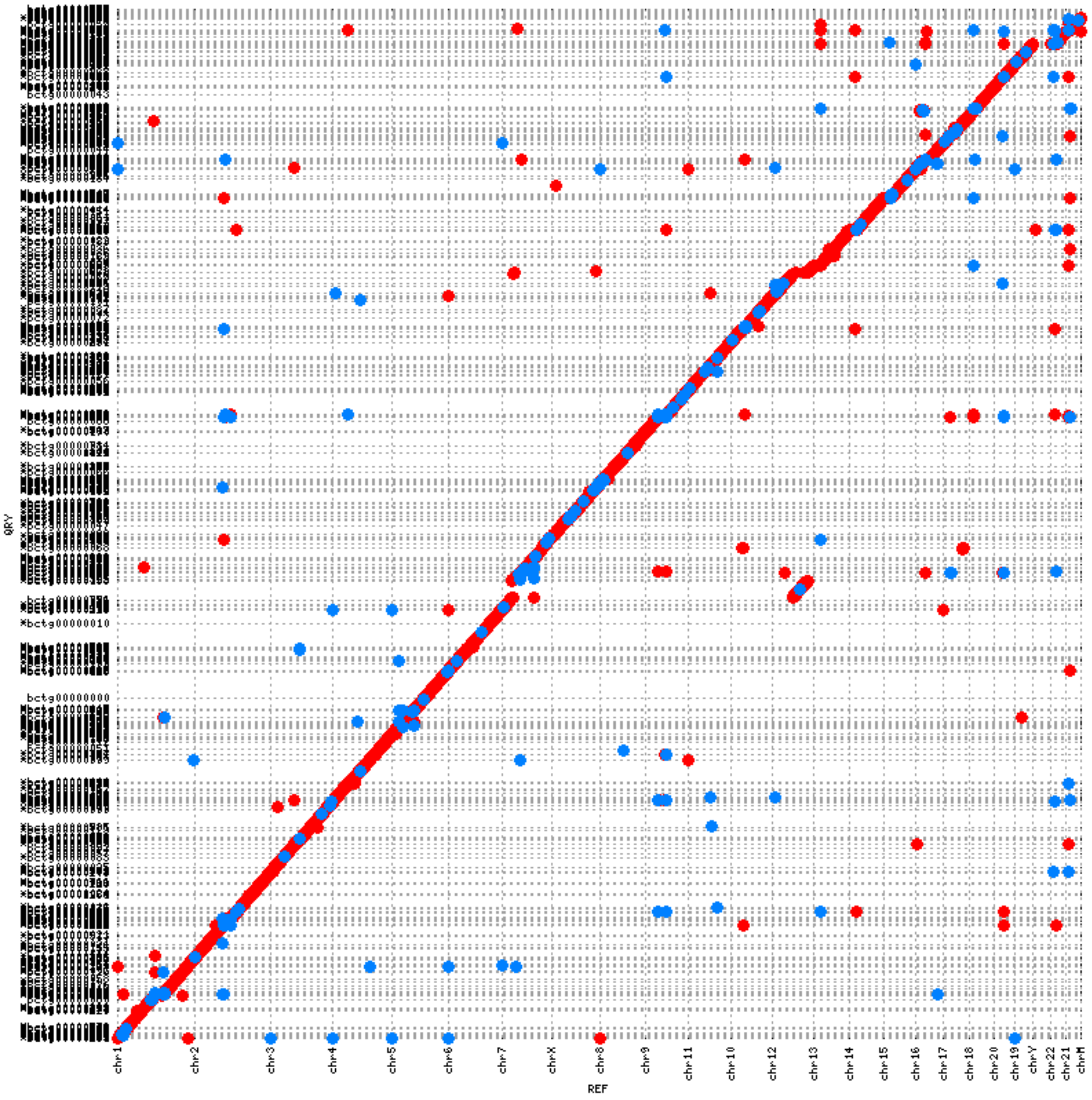

Supplementary Figure 12

Mummerplot of new assembled contigs of NA12878 (rel3,4) Nanopore and hg38 reference genome. An alignment dotplot shows the relationship between the contig assembled using Nanopore (y-axis) and GRCh38 reference genome (x-axis). Contig and chromosome boundaries are displayed as dotted lines (horizontal and vertical, respectively).

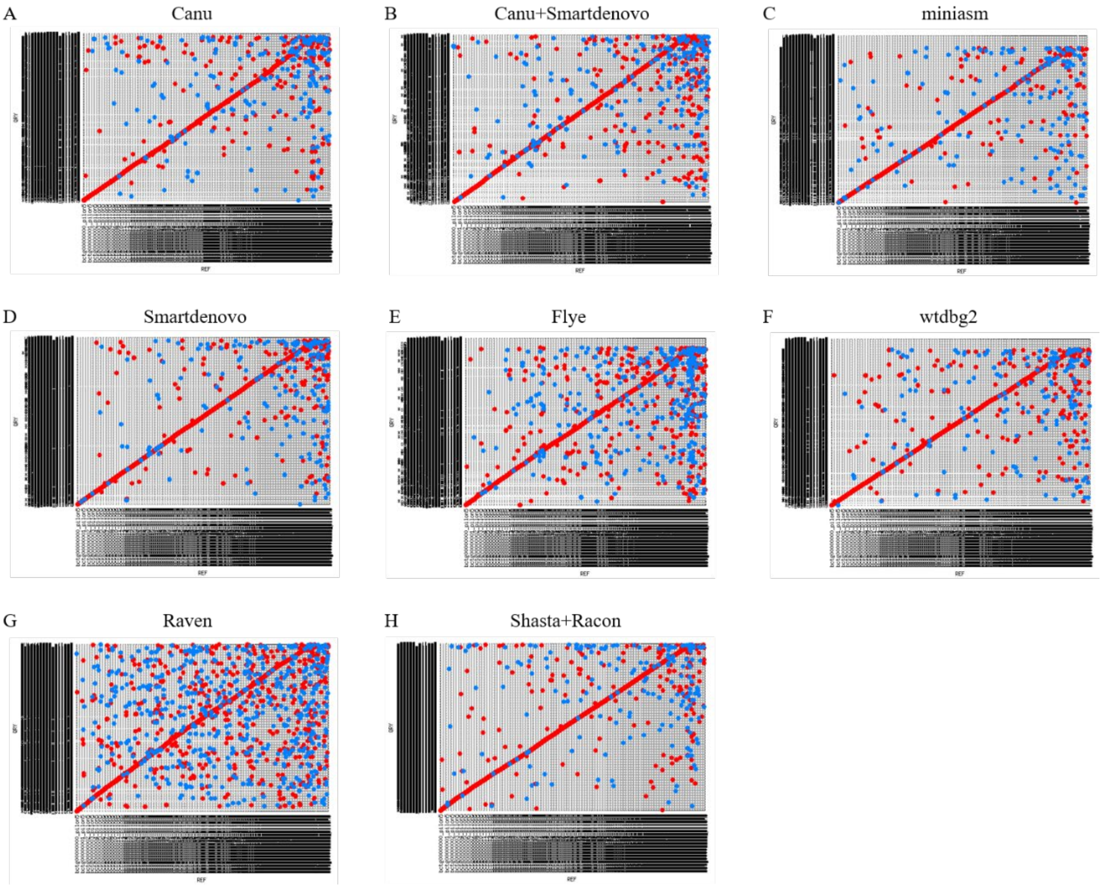

Supplementary Figure 13

Mummerplot of the NECAT contig and *the other assembled* contig from S. pennellii. An alignment dotplot shows the relationship between the contig assembled using Nanopore (y-axis) and S. pennellii reference genome (x-axis). Contig and chromosome boundaries are displayed as dotted lines (horizontal and vertical, respectively).

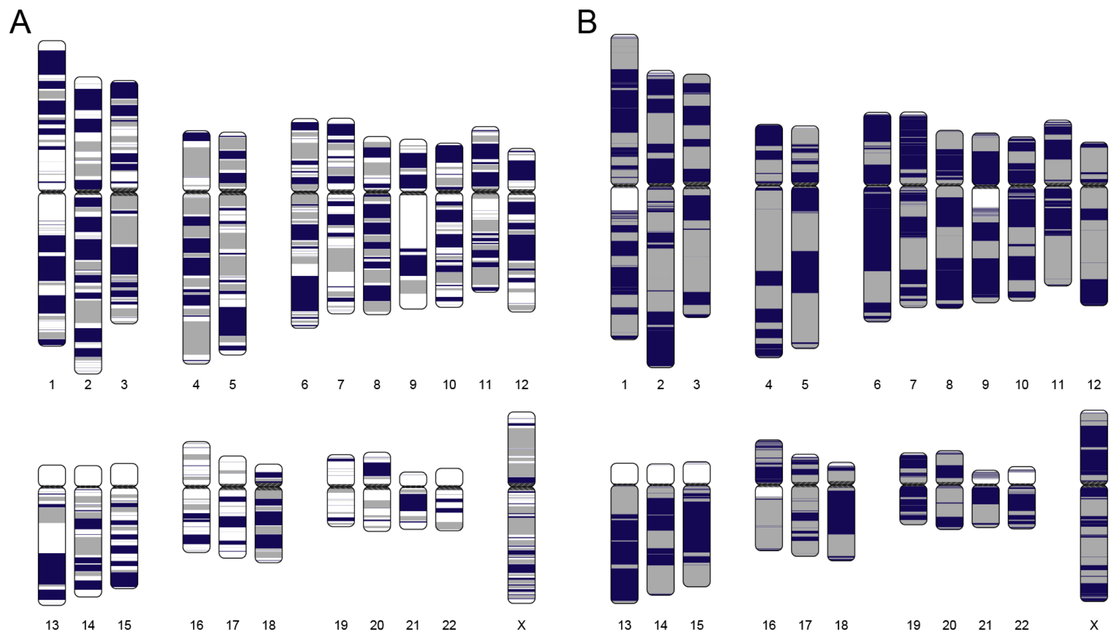

Supplementary Figure 14

Continuity analysis of NECAT and Canu Nanopore *H. sapiens* NA12878 (rel3,4) assembly. (A) NECAT assembly. (B) Canu assembly. Human chromosomes are painted with assembled contigs using ColoredChromosomes package. Alternating shades indicate adjacent contigs (each vertical transition from gray to black represents a contig boundary or alignment breakpoint).

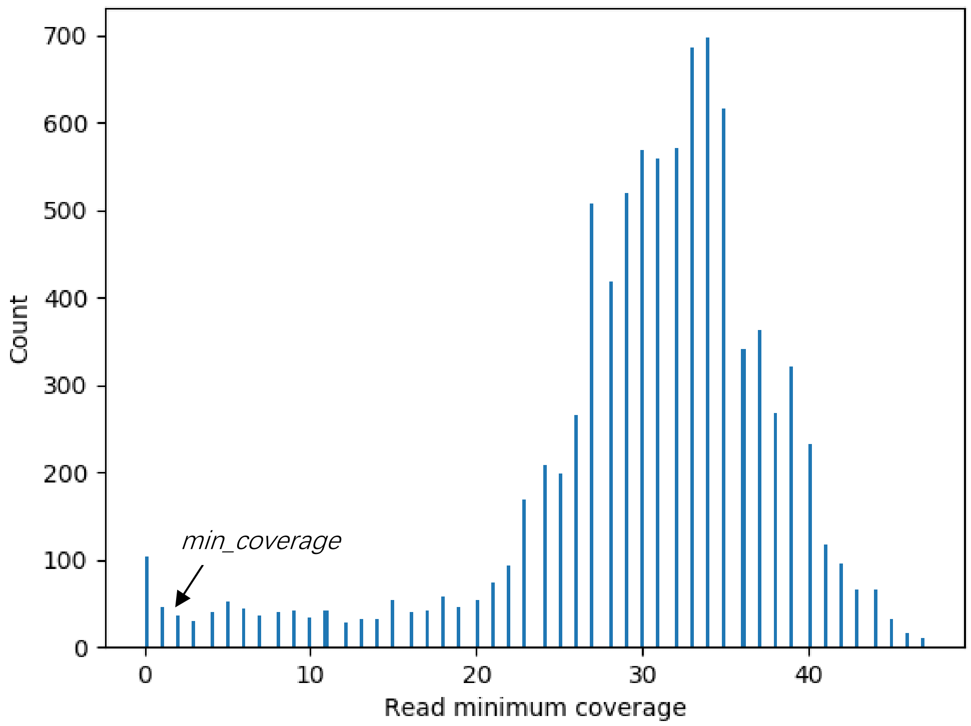

Supplementary Figure 15

Histogram of minimum coverage for the *S. cerevisiae* dataset. $min\_coverage$ is set to the first value not exceeding 30% of the value for the first trough of the histogram of all c_{min}s, where c_{min} is the minimum coverage of all bases.

Supplementary Table 1. Detail information of the nine datasets used in this study.

Datasets	Data resource of LRs	SRA ID of NGS	Reference
E. coli	Ecoli.fasta<br>ecoli.fast5.tgz	SRR072235	K-12 substr. MG1655
S. cerevisiae	yeast.fastq<br>yeast.fast5.tar.gz	SRR5244182	*S. cerevisiae* S288c
D. melanogaster	SRX3676783<br>Dro1.fast5.tar.gz<br>Dro2.fast5.tar.gz	SRR6702604	*D. melanogaster* v6
A. thaliana	ERR2173373<br>Ara.bam	ERR2173372	*A. thaliana* v5
C. reinhardtii	chl.fastq.gz<br>chl.fast5.tar.gz	SRR1734612	*C. reinhardtii* v5.5
O. sativa	rice.fastq.gz	—	O. sativa v4.0
S. pennellii	tomatodata	—	S. pennellii
H. sapiens<br>(NA12878)	rel_3_4 data<br>rel_6_data	ERR194147	Hg38

LRs: long reads; NGS: Next generation sequencing

Supplementary Table 2. Statistical information of the nine datasets used in this study.

Datasets	Base size	Coverage	LR Count	N25	N50	N75	Mean
E. coli	1,481,822,528	322X	164,472	25,243	14,891	8,074	9,010
S. cerevisiae	7,354,232,165	612X	1,814,834	27,799	13,354	4,108	4,052
D. melanogaster	9,064,470,438	66X	1,327,569	18,919	11,853	6,495	6,828
A. thaliana	3,421,779,258	27X	300,071	30,160	20,127	11,544	11,403
C. reinhardtii	16,124,079,751	134X	1,455,141	45,167	27,507	14,469	11,080
O. sativa	67,710,180,969	183X	2,696,991	46,050	33,120	21,676	25,106
S. pennellii	141,704,928,841	160X	11,967,377	21,210	16,611	12,552	11,841
NA12878 (rel3,4)	114,380,310,980	38X	15,599,457	22,232	12,196	7,209	7,332
NA12878(rel6)	132,931,102,331	44X	15,666,888	26,903	13,630	7,984	8,485

HERS: high error subsequences with high sequencing error rates > 50% in 500bp subsequence.

Supplementary Table 3. Statistical information of sequencing error rate for nine datasets.

Datasets	The mean error rate of raw reads	Percentage of reads with HERS and length>10kb
E.coli	17.80%	3.50%
S. cerevisiae	12.00%	5.20%
A. thaliana	20.10%	23.2%
D. melanogaster	16.20%	6.90%
C. reinhardtii	15.00%	10.90%
O. sativa	15.60%	8.10%
S. pennellii	18.49%	8.05%
NA12878(rel3,4)	18.50%	7.80%
NA12878(rel6)	12.08%	5.15%

HERS: high error subsequences with high sequencing error rates > 50% in 1000bp subsequence.

Supplementary Table 4. Comparison of the accuracy of the nine datasets used in this study.

Species	Error rate	Percentage			
		Raw	Canu	CN1	CN2
A. thaliana	1%	0.10%	0.08%	0.13%	0.29%
	2%	0.19%	0.32%	0.82%	3.51%
	3%	0.33%	0.80%	3.07%	15.08%
	4%	0.42%	1.64%	8.86%	17.27%
	5%	0.53%	5.25%	12.79%	9.70%
	6%	0.69%	10.27%	9.60%	6.62%
	7%	0.99%	10.13%	7.06%	5.28%
	8%	1.29%	8.25%	5.83%	4.51%
	9%	1.36%	6.72%	4.91%	3.98%
	10%	1.47%	5.73%	4.37%	3.78%
	10-15%	21.93%	19.98%	17.24%	12.89%
	15-20%	23.94%	13.53%	11.63%	7.01%
	20-15%	19.40%	8.44%	5.88%	3.90%
	25-30%	14.37%	4.27%	3.34%	2.65%
	30-100%	13.00%	4.60%	4.47%	3.52%
E. coli	1%	0.00%	0.00%	0.00%	0.21%
	2%	0.00%	0.02%	0.57%	38.03%
	3%	0.00%	0.12%	13.41%	54.65%
	4%	0.00%	6.49%	39.09%	5.51%
	5%	0.01%	13.82%	27.44%	0.94%
	6%	0.05%	14.93%	10.95%	0.30%
	7%	0.12%	17.18%	4.26%	0.12%
	8%	0.26%	16.45%	1.83%	0.06%
	9%	0.52%	12.85%	0.90%	0.03%
	10%	1.10%	8.23%	0.46%	0.03%
	10-15%	30.91%	9.36%	0.66%	0.05%
	15-20%	37.18%	0.44%	0.19%	0.02%
	20-15%	20.07%	0.07%	0.09%	0.01%
	25-30%	8.53%	0.03%	0.06%	0.01%
	30-100%	1.23%	0.01%	0.10%	0.01%
S. cerevisiae	1%	0.01%	8.93%	22.94%	73.24%

	2%	0.03%	31.27%	32.96%	13.85%
	3%	0.16%	24.28%	17.77%	4.26%
	4%	0.49%	14.81%	9.57%	2.37%
	5%	0.92%	8.01%	4.85%	1.32%
	6%	1.71%	4.28%	2.64%	0.76%
	7%	4.74%	2.42%	1.58%	0.50%
	8%	9.05%	1.53%	1.10%	0.38%
	9%	11.09%	1.02%	0.84%	0.34%
	10%	10.47%	0.67%	0.65%	0.20%
	10-15%	35.62%	1.60%	2.22%	1.02%
	15-20%	16.69%	0.53%	1.15%	0.63%
	20-15%	6.44%	0.25%	0.60%	0.40%
	25-30%	1.41%	0.17%	0.37%	0.25%
	30-100%	1.16%	0.23%	0.75%	0.49%
D. melanogaster	1%	0.41%	1.40%	4.62%	39.13%
	2%	0.44%	20.23%	24.01%	24.44%
	3%	0.43%	20.70%	20.10%	4.65%
	4%	0.47%	9.79%	10.30%	2.03%
	5%	0.55%	5.45%	5.15%	1.78%
	6%	0.73%	3.98%	3.30%	1.84%
	7%	1.35%	3.01%	2.68%	1.43%
	8%	2.83%	2.29%	2.20%	1.03%
	9%	4.91%	1.84%	1.69%	1.34%
	10%	6.62%	1.91%	1.34%	2.51%
	10-15%	32.94%	8.34%	7.01%	9.35%
	15-20%	21.64%	8.52%	8.18%	5.16%
	20-15%	13.97%	6.89%	5.24%	2.89%
	25-30%	7.91%	3.34%	2.46%	1.44%
	30-100%	4.79%	2.31%	1.71%	0.99%
C. reinhardtii	1%	0.40%	0.10%	0.96%	36.91%
	2%	0.24%	9.99%	17.07%	42.08%
	3%	0.18%	35.85%	33.06%	10.98%
	4%	0.16%	20.99%	21.11%	3.56%
	5%	0.18%	9.12%	9.93%	1.65%

	6%	0.39%	4.87%	4.84%	0.94%
	7%	0.69%	3.33%	2.76%	0.59%
	8%	0.75%	2.32%	1.78%	0.42%
	9%	1.11%	1.73%	1.24%	0.33%
	10%	3.28%	1.27%	0.91%	0.25%
	10-15%	61.31%	3.64%	2.41%	0.75%
	15-20%	17.43%	1.91%	1.14%	0.42%
	20-15%	6.35%	1.52%	0.77%	0.39%
	25-30%	3.16%	1.27%	0.66%	0.27%
	30-100%	4.39%	2.10%	1.35%	0.48%
O. sativa	1%	0.05%	0.13%	0.25%	4.85%
	2%	0.06%	1.63%	5.32%	24.66%
	3%	0.09%	11.43%	14.88%	20.55%
	4%	0.12%	15.56%	15.99%	14.56%
	5%	0.17%	15.67%	15.05%	10.00%
	6%	0.27%	12.70%	12.18%	6.73%
	7%	0.43%	9.10%	9.15%	4.28%
	8%	0.65%	6.30%	6.61%	2.49%
	9%	1.14%	4.19%	4.61%	1.33%
	10%	2.47%	2.62%	2.96%	0.77%
	10-15%	42.41%	5.63%	5.16%	3.63%
	15-20%	42.39%	5.85%	3.47%	3.25%
	20-15%	5.46%	5.30%	2.82%	1.69%
	25-30%	2.75%	2.57%	0.88%	0.64%
	30-100%	1.56%	1.33%	0.66%	0.58%
S. pennellii	1%	0.18%	0.10%	0.45%	3.23%
	2%	0.18%	1.92%	3.83%	17.68%
	3%	0.27%	7.76%	8.96%	20.36%
	4%	0.43%	12.46%	12.90%	13.31%
	5%	0.64%	11.80%	12.63%	8.46%
	6%	0.90%	9.45%	10.08%	5.86%
	7%	1.25%	7.37%	7.71%	4.17%
	8%	1.80%	5.55%	5.93%	3.14%
	9%	2.52%	4.15%	4.53%	2.60%

	10%	3.40%	3.16%	3.46%	2.46%
	10-15%	26.81%	15.57%	12.25%	7.85%
	15-20%	25.30%	11.24%	6.90%	4.17%
	20-15%	16.76%	5.23%	3.89%	2.76%
	25-30%	10.10%	2.18%	2.41%	2.05%
	30-100%	9.48%	2.07%	4.09%	1.89%
NA12878(rel3,4)	1%	0.19%	--	0.20%	0.32%
	2%	0.12%	--	0.36%	3.04%
	3%	0.10%	--	2.76%	23.87%
	4%	0.11%	--	11.57%	32.48%
	5%	0.14%	--	19.44%	17.89%
	6%	0.21%	--	19.09%	8.18%
	7%	0.33%	--	14.15%	3.74%
	8%	0.54%	--	9.38%	1.83%
	9%	0.86%	--	6.00%	1.01%
	10%	1.42%	--	3.82%	0.65%
	10-15%	28.36%	--	6.34%	1.54%
	15-20%	34.60%	--	1.80%	1.40%
	20-15%	17.76%	--	2.03%	2.25%
	25-30%	9.13%	--	1.56%	1.20%
	30-100%	6.11%	--	1.50%	0.60%
NA12878(rel6)	1%	0.67%	--	14.22%	43.28%
	2%	0.39%	--	38.31%	27.29%
	3%	0.78%	--	17.05%	3.04%
	4%	2.10%	--	5.48%	1.12%
	5%	4.97%	--	2.18%	0.72%
	6%	8.81%	--	1.08%	0.53%
	7%	10.88%	--	0.71%	0.44%
	8%	10.30%	--	0.58%	0.38%
	9%	8.59%	--	0.52%	0.30%
	10%	6.93%	--	0.44%	0.27%
	10-15%	20.55%	--	1.73%	1.58%
	15-20%	9.88%	--	2.27%	3.87%
	20-15%	6.16%	--	6.54%	9.09%

	25-30%	4.60%	--	4.86%	5.13%
	30-100%	4.39%	--	4.03%	2.97%

Supplementary Table 5. Comparison with assemble-then-correct assemblers

Genome	Pipeline	Assembly Size (Mb)	Contig	NG50 (Kb)	NGA50 (Kb)	MA / local MA	QV (pre- / post-polish)	BUSCO	Total time
E. coli	Ref.	4.6	1	4,642	—	—/—	—/—	—	—
	Miniasm+Racon	4.6	1	4,598	3,919	2/4	17.6/22.2	20.7%	5.0
	Smartdenovo	4.6	1	4,632	3,386	2/3	19.1/22.2	20.0%	40.0
	Wtdbg2	4.5	1	4,495	1,658	3/1	17.0/22.4	17.9%	0.8
	Flye	4.6	1	4,622	3,071	2/2	20.2/22.6	20.2%	630.4
	Raven+Racon	4.6	1	4,599	3,402	2/3	18.9/22.6	20.2%	4.6
	Shasta	4.6	1	4,603	2,924	3/6	18.9/22.5	20.5%	2.7
	NECAT	4.6	1	4,595	3,984	2/3	18.5/22.3	19.8%	2.8
S. cerevisiae	S228C	12.2	17	924	—	—/—	—/—	—	—
	Miniasm+Racon	13.0	33	821	708	39/43	20.9/27.2	97.7%	75.6
	Smartdenovo	12.4	20	937	708	25/37	23.4/28.6	98.4%	97.1
	Wtdbg2	12.1	22	792	701	18/26	22.0/29.5	98.5%	6.3
	Flye	12.3	26	943	706	21/26	21.8/29.0	98.5%	197.8
	Raven	12.5	18	818	714	34/35	24.1/28.7	98.3%	34.9
	Shasta+Racon	12.1	269	927	688	21/24	21.8/29.1	97.4%	17.0
	NECAT	12.3	19	937	708	26/35	23.1/29.0	98.3%	9.3
A. thaliana	TAIR10	119.7	7	23,460	—	—/—	—/—	—	—
	Miniasm+Racon	118.2	69	11,938	559	665/1262	16.4/19.4	98.6%	9.3
	Smartdenovo	116.4	127	3,676	440	370/1201	16.0/19.4	98.6%	78.4
	Wtdbg2	115.3	349	9,840	481	441/1320	15.0/19.6	98.5%	14.4
	Flye	126.6	154	12,043	627	1085/1962	16.8/18.5	98.7%	59.4
	Raven	116.9	25	11,153	601	790/1659	16.7/19.5	98.8%	9.7
	Shasta+Racon	82.5	1071	157	76	205/722	17.3/20.5	82.5%	4.9
	NECAT	122.9	136	11,157	582	886/1304	16.0/18.9	98.8%	47.9
D. melanogaster	dm6	143.7	1870	25,287	—	—/—	—/—	—	—
	Miniasm+Racon	143.9	439	1,496	1,238	906/562	20.1/22.6	91.4%	43.8
	Smartdenovo	138.1	238	4,480	2,915	552/402	20.8/23.0	91.7%	182.4
	Wtdbg2	138.9	872	6,633	4,383	652/301	19.3/22.6	90.4%	26.0
	Flye	139.9	593	11,925	5,129	558/749	21.4/22.5	89.9%	127.9
	Raven	139.1	201	5,914	3,720	723/351	20.7/23.0	91.6%	80.9
	Shasta+Racon	127.6	783	456	445	186/106	21.7/24.2	90.3%	9.8
	NECAT	142.8	277	18,072	6,323	1117/1333	20.2/22.3	92.0%	70.4
C. reinhardtii	Ref. v5.5	111.1	53	7,784	—	—/—	—/—	—	—
	Miniasm+Racon	128.0	215	2,815	588	994/2568	19.5/21.6	98.5%	137.8
	Smartdenovo	112.9	83	3,370	620	748/1681	19.9/22.6	97.2%	1365.3
	Wtdbg2	115.7	344	4,290	711	808/1254	17.9/22.1	97.2%	35.4
	Flye	112.9	65	6,573	831	764/2029	21.6/23.6	98.4%	185.8
	Raven	113.3	49	4,435	774	861/1834	21.3/23.1	98.5%	64.2
	Shasta+Racon	106.9	905	2,099	602	553/1490	21.3/23.1	95.5%	36.5
	NECAT	113.4	54	6,169	732	831/2273	19.8/22.4	98.0%	101.8
O. sativa	Ref.v4.0	382.8	15	30,829	—	—/—	—/—	—	—
	Miniasm+Racon	393.0	240	9,750	3,078	625/7104	15.2/16.0	59.0%	933.1
	Smartdenovo	379.4	352	1,889	1,363	467/7987	16.5/16.0	59.6%	3564.9
	Wtdbg2	394.6	2554	2,432	1,272	396/10878	14.6/15.8	55.9%	154.3
	Flye	380.7	249	3,552	2,213	573/1742	16.4/16.3	59.2%	817.6
	Raven	374.8	212	3,670	2,109	563/3738	16.2/16.3	60.1%	344.6
	Shasta+Racon	345.8	3278	340	296	248/4515	15.9/16.7	58.2%	161.1
	NECAT	373.1	120	9,650	3,311	479/4873	16.0/16.3	58.4%	517.2
S. pennellii	Ref.	915.6	899	2,522	—	—/—	—/—	—	—
	Miniasm	977.8	2704	1,903	1	694/56129	—/18.3	83.8%	—
	Smartdenovo	955.3	1901	1,108	574	3544/14483	—/20.2	97.0%	—
	Wtdbg2	934.3	4986	1,228	632	3258/9948	15.2/16.9	93.6%	439.0
	Flye	1,026.0	3180	1,971	651	8504/10726	16.0/18.5	96.7%	3590.8
	Raven	1,019.7	3287	609	368	10254/12375	16.2/19.9	94.0%	1119.1
	Shasta+Racon	748.7	9712	115	100	1909/3528	16.8/21.1	96.2%	344.7
	NECAT	991.8	1344	4,802	992	5813/12592	15.2/17.3	95.5%	3233.7
NA12878 (rel6)	Ref.38	3,272	639	145,139	—	—/—	—/—	—	—
	Flye	2,867	3309	28,407	16,640	4054/7258	22.9/24.2	74.6%	2500.0
	NECAT	2,847	1047	20,913	13,441	948/1467	23.1/24.4	74.5%	9418.8

'Assembly size' is the total number of base pairs in all contigs generated by assemblers. 'NG50' indicates that 50% of reference

genome size was contained in contigs having length ≥N. 'NGA50' is NG50 of aligned blocks that contigs are broken into at misassembly breakpoints. 'MA / local MA' are the numbers of misassemblies and local misassemblies evaluated by QUAST. 'QV' is defined as $10 \times \log_{10}(\frac{100kbp}{\#\ mismatches\ per\ 100\ kbp + \#\ indels\ per\ 100\ kbp})$, where '*# mismatches per 100 kbp*' and '*# indels per 100 kbp*' are evaluated by QUAST. 'BUSCO' is gene completeness evaluated by BUSCO. All the pipelines were tested on the same computer with 2.0 GHz CPU and 3T GB RAM of memory. For the first six datasets, we ran all the pipelines on our computer with 32 threads; the total computational time are recorded. For *S. pennellii* and human dataset, we ran the pipelines on our computer with 64 threads, and total computational time were recorded. The assembly results of Miniasm and Smartdenove on the dataset *S. pennellii* were from https://www.plabipd.de/portal/solanum-pennellii. The assembly results and total time of Flye on the dataset NA12878 (rel6) was acquired from https://github.com/fenderglass/Flye.

Supplementary Table 6. SNP and INDEL statistics between assembly genome and reference genome.

Species	Software	SNPs	Indels		Cover (%)
			<=10bp	>10bp	
E. coli	Canu	9374	1	18	99.35
	Canu+Smartdenovo	9170	0	4	99.39
	Smartdenovo	8663	0	6	99.31
	Miniasm+Racon	10116	0	6	99.33
	wtdbg2	7926	0	4	99.39
	Flye	8590	0	3	99.42
	Raven	8898	0	3	99.41
	Shasta+Racon	9250	1	5	99.38
	NECAT	10248	0	11	99.37
S. cerevisiae	Canu	9297	7	33	99.88
	Canu+Smartdenovo	9010	4	30	99.91
	Smartdenovo	9699	8	41	99.65
	miniasm+Racon	9508	23	31	99.43
	wtdbg2	9243	7	31	99.82
	Flye	9199	8	31	99.92
	Raven	9175	15	34	99.90
	Shasta+Racon	9311	31	45	98.50
	NECAT	9142	4	38	99.92
D. melanogaster	Canu	24811	22	266	98.96
	Canu+Smartdenovo	31359	9	281	99.16
	Smartdenovo	40735	5	251	98.92
	miniasm+Racon	44233	54	285	98.10
	wtdbg2	27297	24	215	98.87
	Flye	26494	20	195	99.03
	Raven	36549	21	224	98.46
	Shasta+Racon	30080	23	213	98.70
	NECAT	29113	10	345	99.30
A. thaliana	Canu	457782	456	2880	98.98
	Canu_smartdenovo	462818	42	3413	99.32
	Smartdenovo	461263	37	3372	99.02

	miniasm+Racon	463272	467	2982	97.04
	wtdbg2	457763	458	2976	99.19
	Flye	463692	465	2988	99.34
	Raven	462823	465	3001	98.58
	Shasta+Racon	328323	335	1960	98.55
	NECAT	463859	45	3427	99.31
C. reinhardtii	Canu	39984	324	1580	99.40
	Canu_smartdenovo	39832	13	1630	99.60
	Smartdenovo	47750	12	1655	99.40
	miniasm+Racon	48898	301	1386	99.07
	wtdbg2	48849	283	1372	99.21
	Flye	39996	302	1388	99.69
	Raven	40500	305	1401	99.49
	Shasta+Racon	46545	295	1364	98.81
	NECAT	45218	11	1812	99.54

Supplementary Table 7. Number of TEs in Flybase.

Method	Contain in a contig		*roo*		*Juan*	
	Total	Perfect	Total	Perfect	Total	Perfect
Canu	5304	3970	131	95	11	11
Canu+Smartdenovo	5292	3916	132	115	11	11
Miniasm+Racon	5234	3994	128	89	9	9
Smrtdenovo	5312	3998	135	106	11	11
Flye	5268	3840	131	93	11	11
Wtdbg2	5156	3831	130	95	11	11
Raven	5269	3943	130	72	11	11
Shasta+Racon	5042	4063	113	69	11	11
NECAT	5,304	4001	134	118	11	11

Total and Perfect refer to all the identified TE numbers and the number of TEs with more than 99% Pct_ident from the final report.

Supplementary Table 8. Chromosome number identified based on the alignment of telomeric repeats

Method	All	Pair_end telomere		Single_end telomere
		Identified in a single contigs	Identified in two contigs	
Canu	16	13	1	2
Canu+Smartdenovo	16	14	1	1
Miniasm+Racon	16	13	1	2
Smartdenovo	16	14	1	1
Flye	16	14	1	1
Wtdbg2	16	3	8	5
Raven	16	14	1	1
Shasta+Racon	16	13	1	2
NECAT	16	14	1	1

"All" indicates the total identified number of chromosome, "identified in a single contig" is the number of chromosomes in which the telomeric repeats are mapped on both the left and right ends in a single contig.

Supplementary Table 9. Comparison with hybrid pipelines

Genome	Pipeline	Assembly Size (Mb)	Contig	NG50 (Kb)	NGA50 (Kb)	MA / local MA	QV	Correct/Contig /Total time
E. coli	Ref.	4.6	1	4,642	—	—/—	—	—/—/—
	Canu+Smartdenovo	4.6	1	4,630	3,287	3/2	18.6	26.1/8.0/34.1
	Canu-Flye	4.6	1	4,606	3,943	2/2	19.6	26.1/1.8/27.9
	Canu+NECAT	4.6	1	4,595	2,451	2/16	17.2	26.1/1.1/27.2
	NECAT+Canu	4.6	1	4,635	3,362	2/2	18.5	1.6/37.9/39.5
	NECAT+Smartdenovo	4.6	1	4,637	3,291	3/2	18.6	1.6/6.5/8.1
	NECAT+Flye	4.6	1	4,599	1,626	4/2	19.0	1.6/2.1/3.7
	NECAT	4.6	1	4,595	3,984	2/3	18.5	1.6/1.2/2.8
S. cerevisiae	S228C	12.2	17	924	—	—/—	—	—/—/—
	Canu+Smartdenovo	12.4	19	815	705	34/29	22.7	493.3/38.4/531.7
	Canu-Flye	12.4	26	939	710	27/31	24.5	493.3/28.7/522.0
	Canu+NECAT	12.4	21	816	705	38/49	21.8	493.3/2.8/496.1
	NECAT+Canu	12.5	20	936	708	28/34	23.0	4.4/257.0/261.4
	NECAT+Smartdenovo	12.3	17	816	676	20/24	23.3	4.4/29.9/34.3
	NECAT+Flye	12.2	16	940	706	22/22	22.2	4.4/11.6/16.0
	NECAT	12.3	19	937	708	26/35	23.1	4.4/4.9/9.3
A. thaliana	TAIR10	119.7	7	23,460	—	—/—	—	—/—/—
	Canu+Smartdenovo	115.6	44	11,071	527	576/1170	15.9	193.1/125.9/319.0
	Canu-Flye	117.2	124	7,157	543	628/1107	16.4	193.1/32.4/225.5
	Canu+NECAT	110.3	280	5,762	511	356/1372	15.2	193.1/15.5/208.6
	NECAT+Canu	120.1	66	11,022	575	835/1235	16.3	19.8/841.9/861.7
	NECAT+Smartdenovo	117.6	57	6,314	582	725/1168	16.5	19.8/101.8/121.6
	NECAT+Flye	118.9	62	13,170	619	952/1381	16.6	19.8/31.1/50.9
	NECAT	122.9	136	11,157	536	886/1304	16.0	19.8/28.0/47.9
D. melanogaster	dm6	143.7	1870	25,287	—	—/—	—	—/—/—
	Canu+Smartdenovo	135.8	162	14,456	6,473	587/333	20.8	289.6/294.4/584.0
	Canu-Flye	136.1	261	3,519	2,497	544/268	23.1	289.6/80.2/369.8
	Canu+NECAT	139.3	291	16,995	7,154	687/674	18.9	289.6/24.0/313.6
	NECAT+Canu	150.4	496	4,872	4,179	1640/1738	20.2	37.7/3434.4/3472.0
	NECAT+Smartdenovo	134.5	123	12,674	9,663	488/295	21.4	37.7/196.3/234.0
	NECAT+Flye	137.3	246	7,369	5,100	578/720	21.1	37.7/69.1/106.7
	NECAT	142.8	277	18,072	6,323	1117/1333	20.2	37.7/32.7/70.4
C. reinhardtii	Ref. v5.5	111.1	53	7,784	—	—/—	—	—/—/—
	Canu+Smartdenovo	109.7	46	4,498	713	655/1629	20.1	950.4/816.0/1766.4
	Canu-Flye	111.7	54	4,149	756	754/1946	21.4	950.4/126.5/1076.9
	Canu+NECAT	110.9	77	4,435	682	650/2125	18.1	950.4/29.1/979.5
	NECAT+Canu	118.9	100	5,317	687	934/2515	20.0	54.8/8299.1/8353.9
	NECAT+Smartdenovo	112.9	72	2,569	622	704/1583	20.4	54.8/690.7/745.5
	NECAT+Flye	112.2	37	6,712	746	770/1947	20.9	54.8/119.8/174.6
	NECAT	113.4	54	6,169	726	831/2273	19.8	54.8/47.0/101.8
NA12878(rel6)	Ref38	3,272	639	145,139	—	—/—	—	—/—/—
	NECAT+Flye	2,844	820	33,800	16,778	850/1017	21.2	2518.4/1599.9/4118.3
	Flye	2,867	3309	28,407	16,640	4054/7258	22.9	—/—/2500
	NECAT	2,847	1047	20,913	13,441	948/1467	23.1	2518.4/6900.4/9418.8

'Assembly size' is the total number of base pairs in all contigs generated by assemblers. 'NG50' indicates that 50% of reference genome size was contained in contigs having length ≥N. 'NGA50' is NG50 of aligned blocks that contigs are broken into at misassembly breakpoints. 'MA / local MA' are the numbers of misassemblies and local missassemblies evaluated using QUAST. 'QV' is defined as $10 \times \log_{10}(\frac{100kbp}{\#\ mismatches\ per\ 100\ kbp + \#\ indels\ per\ 100\ kbp})$, where '*# mismatches per 100 kbp*' and '*# indels per 100 kbp*' are evaluated by QUAST. All the pipelines were tested on the same computer with 2.0 GHz CPU and 3T GB RAM of memory. We ran all the pipelines on our computer with 32 threads for the first five datasets and with 64 threads for the human dataset; the correction and contig computational time of the pipelines were recorded. The assembly

results and total time of Flye on the dataset NA12878 (rel6) was acquired from https://github.com/fenderglass/Flye.

Reference

1. Li, H. Minimap and miniasm: fast mapping and de novo assembly for noisy long sequences. *Bioinformatics* **32**, 2103 (2015).
2. Ruan, J. & Li, H. Fast and accurate long-read assembly with wtdbg2. *Nature Methods* (2019).
3. Kolmogorov, M., Yuan, J., Lin, Y. & Pevzner, P.A. Assembly of long, error-prone reads using repeat graphs. *Nature Biotechnology* **37**, 540-546 (2019).
4. Vaser, R., Sović, I., Nagarajan, N. & Šikić, M. (2020).
5. Shafin, K. et al. Nanopore sequencing and the Shasta toolkit enable efficient de novo assembly of eleven human genomes. *Nature Biotechnology* (2020).
6. Gurevich, A., Saveliev, V., Vyahhi, N. & Tesler, G. QUAST: quality assessment tool for genome assemblies. *Bioinformatics* **29**, 1072-1075 (2013).
7. Vaser, R., Sović, I., Nagarajan, N. & Šikić, M. Fast and accurate de novo genome assembly from long uncorrected reads. *Genome Res* **27**, 737-746 (2017).
8. Loman, N.J., Quick, J. & Simpson, J.T. A complete bacterial genome assembled de novo using only nanopore sequencing data. *Nature Methods* **12**, 733-735 (2015).
9. Walker, B.J. et al. Pilon: an integrated tool for comprehensive microbial variant detection and genome assembly improvement. *Plos One* **9**, e112963 (2014).
10. Kingan, S. et al. A High-Quality De novo Genome Assembly from a Single Mosquito Using PacBio Sequencing. *Genes*.
11. Vaser, R., Sović, I., Nagaranjan, N. & Šikić, M. Fast and accurate de novo genome assembly from long uncorrected reads. *Genome Research* **27**, 737 (2017).
12. Kurtz, S. et al. Versatile and open software for comparing large genomes. *Genome Biology* **5**, R12 (2004).
13. Salzberg, S.L. et al. GAGE: A critical evaluation of genome assemblies and assembly algorithms. *Genome research* **22**, 557-567 (2012).
14. Schmidt, M.H.W. et al. De Novo Assembly of a New Solanum pennellii Accession Using Nanopore Sequencing. *The Plant Cell* **29**, 2336 (2017).
15. Hoskins, R.A. et al. The Release 6 reference sequence of the Drosophila melanogaster genome. *Genome Research* **25**, 445-458 (2015).
16. Hoskins, R.A. et al. Sequence finishing and mapping of Drosophila melanogaster heterochromatin. *Science* **316**, 1625-1628 (2007).
17. Kaminker, J.S. et al. The transposable elements of the Drosophila melanogaster euchromatin: a genomics perspective. *Genome Biology* **3**, 1-20 (2002).
18. Fu, Y. et al. N6-Methyldeoxyadenosine Marks Active Transcription Start Sites in Chlamydomonas. *Cell* **161**, 879-892 (2015).
19. Michael, T.P. et al. High contiguity Arabidopsis thaliana genome assembly with a single nanopore flow cell. *Nature Communications* **9**, 541 (2018).
20. Solares, E.A., Chakraborty, M., Miller, D.E., Kalsow, S. & Hawley, R.S. Rapid Low-Cost Assembly of the Drosophila melanogaster Reference Genome Using Low-Coverage, Long-Read Sequencing. *G3: Genes|Genomes|Genetics* **8**, g3.200162.202018 (2018).
21. Jain, M. et al. Nanopore sequencing and assembly of a human genome with ultra-long reads. *Nature Biotechnology* **36**, 338-345 (2018).
22. Staff, S.R.A.S. Using the SRA Toolkit to convert .sra files into other formats. (2011).

23. Shen, W., Le, S., Li, Y. & Hu, F. SeqKit: A Cross-Platform and Ultrafast Toolkit for FASTA/Q File Manipulation. *PloS one* **11**, e0163962 (2016).
24. Konstantin, B. et al. Assembling large genomes with single-molecule sequencing and locality-sensitive hashing. *Nature Biotechnology* **33**, 623-630 (2015).
25. Tischler, G. & Myers, E.W. Non Hybrid Long Read Consensus Using Local De Bruijn Graph Assembly. *bioRxiv* (2017).
26. Koren, S. et al. Hybrid error correction and de novo assembly of single-molecule sequencing reads. *Nature Biotechnology* **30**, 693-700 (2012).
27. Li, H. Minimap2: pairwise alignment for nucleotide sequences. *Bioinformatics* **34** (2017).
28. Koren, S. et al. Canu: scalable and accurate long-read assembly via adaptive k-mer weighting and repeat separation. *Genome Research* **27**, 722 (2017).
29. Schmidt, M.H. et al. De novo Assembly of a New Solanum pennellii Accession Using Nanopore Sequencing. *Plant Cell* **29**, 2336 (2017).
30. Ruan, J. & Li, H. Fast and accurate long-read assembly with wtdbg2. *Nature Methods* **17**, 1-4 (2020).
31. Kolmogorov, M., Yuan, J., Lin, Y. & Pevzner, P.A. Assembly of long, error-prone reads using repeat graphs. *Nature Biotechnology* (2019).
32. Sedlazeck, F.J. et al. Accurate detection of complex structural variations using single-molecule sequencing. *Nature Methods* **15** (2018).
33. Layer, R.M., Chiang, C., Quinlan, A.R. & Hall, I.M. LUMPY: a probabilistic framework for structural variant discovery. *Genome Biology* **15**, R84 (2014).
34. Schmidt, M.H. et al. De Novo Assembly of a New Solanum pennellii Accession Using Nanopore Sequencing. *The Plant cell* **29**, 2336-2348 (2017).
35. Seppey, M., Manni, M. & Zdobnov, E.M. BUSCO: Assessing Genome Assembly and Annotation Completeness. *Methods in molecular biology (Clifton, N.J.)* **1962**, 227-245 (2019).
36. Phillippy, A.M., Schatz, M.C. & Pop, M. Genome assembly forensics: finding the elusive mis-assembly. *Genome Biology* **9**, 1-13 (2008).
37. Salzberg, S.L. et al. GAGE: A critical evaluation of genome assemblies and assembly algorithms. (2012).
