# Supplementary material for: Efficient assembly of nanopore reads via highly accurate and intact error correction
Source: Nat Commun. 2021 Jan 4;12:60. doi: 10.1038/s41467-020-20236-7 (PMC7782737; doi:10.1038/s41467-020-20236-7)
Supplement: Supplementary file 3 — Description of Additional Supplementary Files [file 41467_2020_20236_MOESM3_ESM.pdf]

Description of Additional Supplementary Files

File Name: Supplementary Data 1.

Description: Structural variants identified using WERI assembly, raw Nanopore long reads and Illumina short reads.

Sheet1: WERI: Structural variants identified in WERI assembly aligned hg38 (>10 bp);

Sheet2: NGS: Structural variants identified in hg38 from Illumina short reads (NGS) data;

Sheet3: TGS: Structural variants identified in hg38 from Nanopore long reads (TGS) data;

Sheet4: TGS-NGS: Consistent structural variants identified from Nanopore long reads (TGS) and Illumina short reads (NGS);

Sheet5: WERI-TGS: Consistent structural variants identified from WERI assembly and TGS;

Sheet6: WERI-NGS: Consistent structural variants identified from WERI assembly and NGS;

Sheet7: WERI-NGS-TGS-overlap: Consistent structural variants identified from WERI assembly, NGS and TGS;

Sheet8: Unique SV for TGS: Unique structural variants identified from TGS.

File Name: Supplementary Data 2.

Description: Genes associated with the identified structural variants identified using WERI assembly, raw Nanopore long reads and Illumina short reads.

Sheet1: WERI_TGS_SV_gene: Genes with structural variants (SVs) identified in WERI assembly from Nanopore long reads (TGS);

Sheet2: NGS_SV_gene: Genes with structural variants identified in WERI assembly from Illumina short reads (NGS);

Sheet3: WERI_TGS_associated_gene: 209 SVs-genes associated with retinoblastoma in WERI assembly from TGS;

Sheet4: overlap_gene: Consistent genes with SVs between WERI-TGS and WERI-NGS;

Sheet5: unique_gene_for_WERI_TGS: Unique genes with SVs identified in WERI assembly from TGS.
